# Identification and functional analyses of host factors interacting with the 17-kDa protein of Barley yellow dwarf virus-GAV

**DOI:** 10.1038/s41598-021-87836-1

**Published:** 2021-04-19

**Authors:** Siyu Chen, Xiaoyu Han, Lingling Yang, Qinglun Li, Yajuan Shi, Honglian Li, Linlin Chen, Bingjian Sun, Yan Shi, Xue Yang

**Affiliations:** College of Plant Protection, Henan Agricultural University, Zhengzhou, 450002 China

**Keywords:** Biotic, Virus-host interactions

## Abstract

Barley yellow dwarf viruses (BYDVs) cause significant economic losses on barley, wheat, and oats worldwide. 17-kDa protein (17K) of BYDVs plays a key role in viral infection in plants, whereas the underlying regulation mechanism of 17K in virus infection remains elusive. In this study, we determined that 17K of BYDV-GAV, the most common species found in China in recent years, was involved in viral pathogenicity. To identify the host factors interacting with 17K, the full length coding sequence of 17K was cloned into pGBKT7 to generate the bait plasmid pGBKT7-17K. 114 positive clones were identified as possible host factors to interact with 17K through screening a tobacco cDNA library. Gene ontology enrichment analysis showed that they were classified into 35 functional groups, involving three main categories including biological processes (BP), cellular components (CC), and molecular functions (MF). Kyoto Encyclopedia of Genes and Genome (KEGG) analysis indicated the acquired genes were assigned to 49 KEGG pathways. The majority of these genes were involved in glyoxylate and dicarboxylate metabolism, carbon fixation in photosynthetic organisms, and glycolysis/gluconeogenesis. The interactions between 17K and the 27 proteins with well-documented annotations were verified by conducting yeast two-hybrid assays and 12 of the 27 proteins were verified to interact with 17K. To explore the putative function of the 12 proteins in BYDV-GAV infection, the subcellular localization and expression alterations in the presence of BYDV-GAV were monitored. The results showed that, under the condition of BYDV-GAV infection, RuBisCo, POR, and PPD5 were significantly up-regulated, whereas AEP and CAT1 were significantly down-regulated. Our findings provide insights into the 17K-mediated BYDV-GAV infection process.

## Introduction

Barley yellow dwarf viruses (BYDVs), a group of viral pathogens that belong to the genus Luteovirus or Polerovirus or are unassigned to a genus (family Luteoviridae), cause significant economic losses on barley, wheat, and oats worldwide^1^. BYDVs are transmitted by aphids in a persistent, circulative, and non-propagative manner and phloem-limited. BYDVs-diseased plants always exhibit typical disease symptoms such as yellowing of leaves and stunting. In China, BYDVs have been classified into four isolates (GPV, GAV, PAV, and RMV) based on the specificity of vectors and serological properties^2–5^ and BYDV-GAV is the most common species in recent years^6^.

The BYDV-GAV is about 5.7 kb in length and comprises seven open reading frames (ORFs), which encode P1, P2, P3, P3a, P4, P5, and P6, respectively. They are involved in viral replication (P1 and P2), virion assembly (P3, the coat protein [CP]), virus spread (P3, P3a, and P4), transmission by the aphid vector (P5), and suppression of RNA silencing (P4 and P6)^2,7–12^. Luteovirus P4 encodes a 17-kDa protein (17K) that functions in viral movement, suppression of host RNA silencing, and viral pathogenesis^8,12,13^. For instance, BYDV-GAV 17K disrupts mitosis by interrupting the function of Wee1-Cdc25-CDKA/Cdc2 via direct protein–protein interactions^12^. But, how 17K assists virus movement, suppresses host antiviral RNA silencing and promotes virus infection remains largely unknown.

In this study, we determined the function of 17K in viral pathogenicity. We identified the host factors interacting with 17K by screening a tobacco cDNA library, and the interactions between 12 host factors and 17K were confirmed by Y2H assays. We further detected the subcellular localization of the interacting host factors and the expression of the interacting host factors after BYDV-GAV infection. Our study may help to comprehensively understand the pathogenesis of BYDV-GAV infection.

## Results

### 17K enhances viral pathogenicity in a heterologous virus expression system

**Figure 1:**
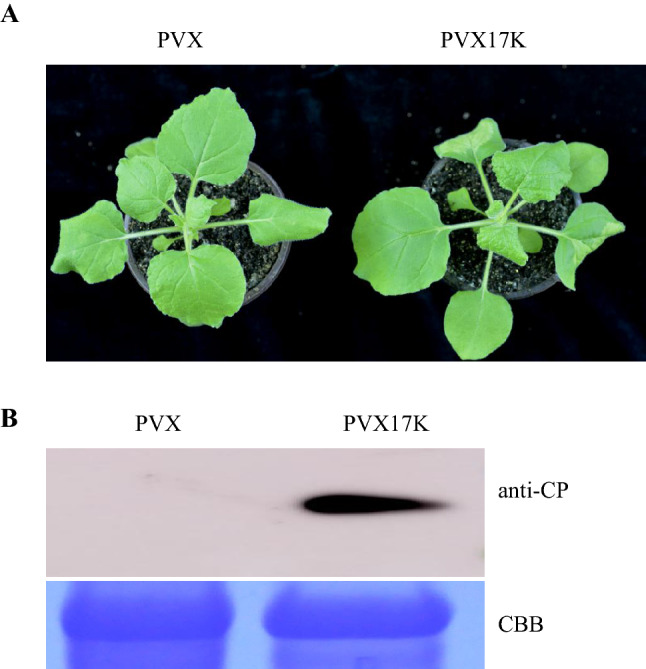
17K promoted PVX infection in *N. benthamiana*. (**A**) Disease symptoms exhibited in *N. benthamiana* leaves inoculated with PVX or PVX17K. (**B**) PVX accumulation in the PVX- or PVX17K-inoculated *N. benthamiana* leaves were detected by conducting Western blotting analysis with anti-CP antibodies. Coomassie Brilliant Blue (CBB) staining of the large subunit of RuBisCo served as loading controls.

To test whether 17K is associated with viral pathogenicity, the protein was expressed in *Nicotiana benthamiana* (*N. benthamiana*) using a PVX-based vector. At 5 days post-infiltration (dpi), the systemic leaves of PVX17K plants showed obvious mosaic symptoms, whereas those treated with PVX showed no symptoms (Fig. 1A). PVX accumulation in PVX17K plants at 5 dpi was indicated by performing Western blotting with PVX CP antibodies, whereas no signal was observed in the PVX control (Figs. 1B, S1). These results suggest that 17K enhances viral pathogenicity of PVX. Additionally, silencing suppressor activity of 17K was tested using a *N. benthamiana* 16c system, and strong fluorescence was observed at 3 dpi in the combinations GFP + P19 and GFP + 17K, indicating silencing suppressor activity of 17K (Fig. S2).

### Subcellular localization pattern of BYDV-GAV 17K

**Figure 2:**
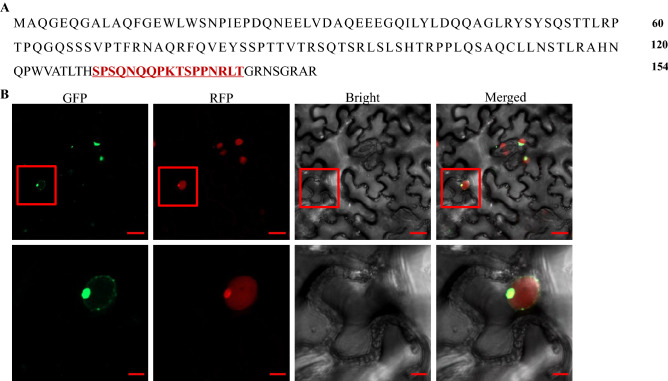
Subcellular localization of 17K in *N. benthamiana* epidermal cells. (**A**) Online analysis of the 17K nuclear localization signal using PortII (https://psort.hgc.jp/form2.html) and seqNLS (http://mleg.cse.sc.edu/seqNLS). The predicted nuclear localization signal sequence is colored in red and underlined. (**B**) Localization of 17K in *N. benthamiana* epidermal cells. GFP-tagged 17K (17K-GFP) was expressed *in planta*. Confocal images were taken at 2 dpi. Bars represent 20 μm and 5 μm, respectively.

Using PortII (https://psort.hgc.jp/form2.html) and seqNLS (http://mleg.cse.sc.edu/seqNLS/), 17K was predicted to be localized in the nucleus and would thus show a nuclear localization signal (Fig. 2A). To illustrate the subcellular localization of BYDV-GAV 17K, 17K-GFP was measured in epidermal cells of *N. benthamiana* plants. The results showed that the green fluorescent signal of 17K-GFP merged with the red fluorescent signal of H2B-RFP, indicating the nuclear localization of BYDV-GAV 17K (Fig. 2B). Our finding is consistent with a previous study^12^. Notably, we found that 17K was monitored mainly at the nuclear envelope (Fig. 2B).

### Identification of 17K-interacting host factors by screening a tobacco library

**Figure 3:**
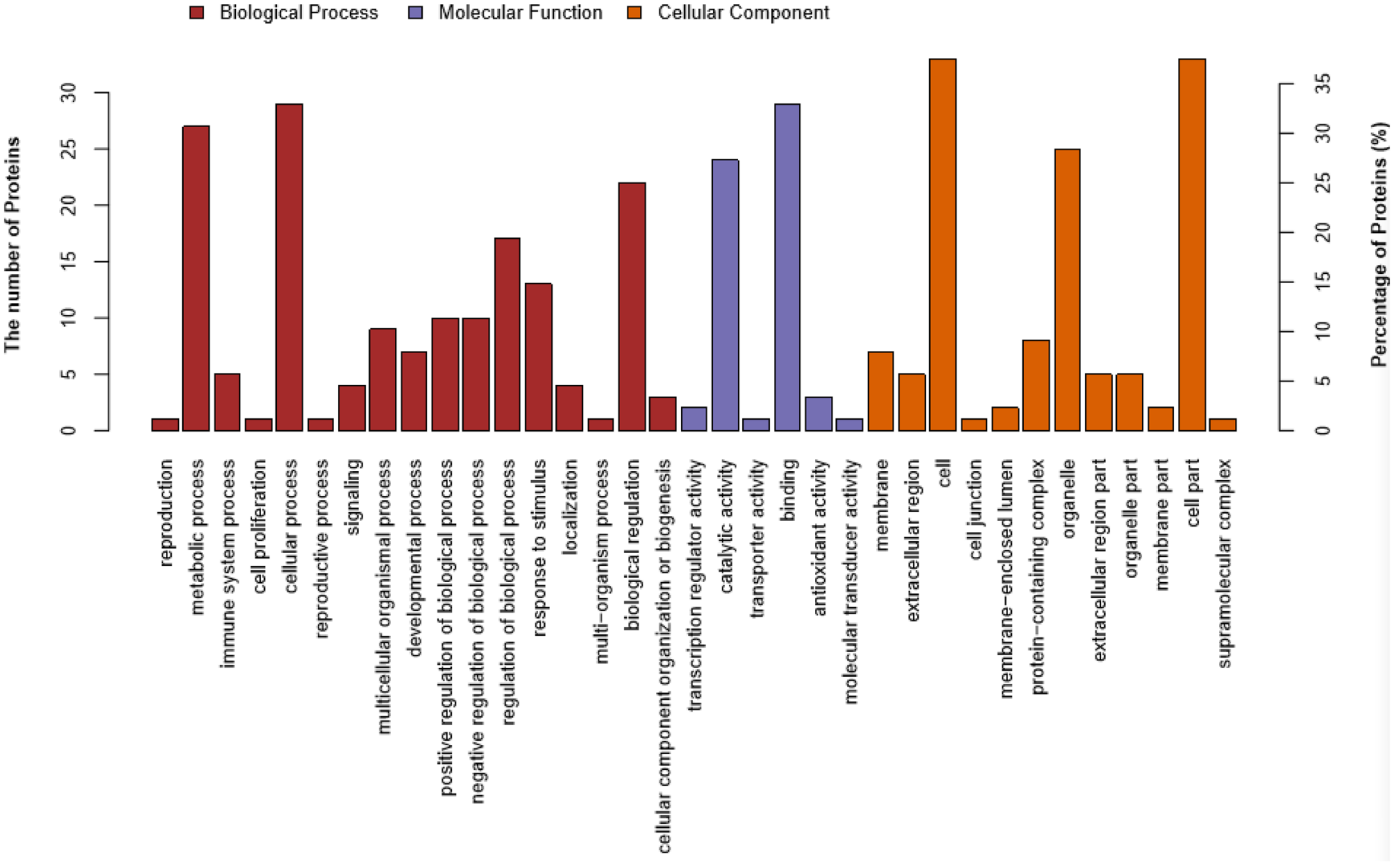
Function categorization of the acquired clones by GO analysis. GO analysis of the screened 17K-interacting genes. The genes were divided into three categories: cellular component, biological process, and molecular function genes.

**Figure 4:**
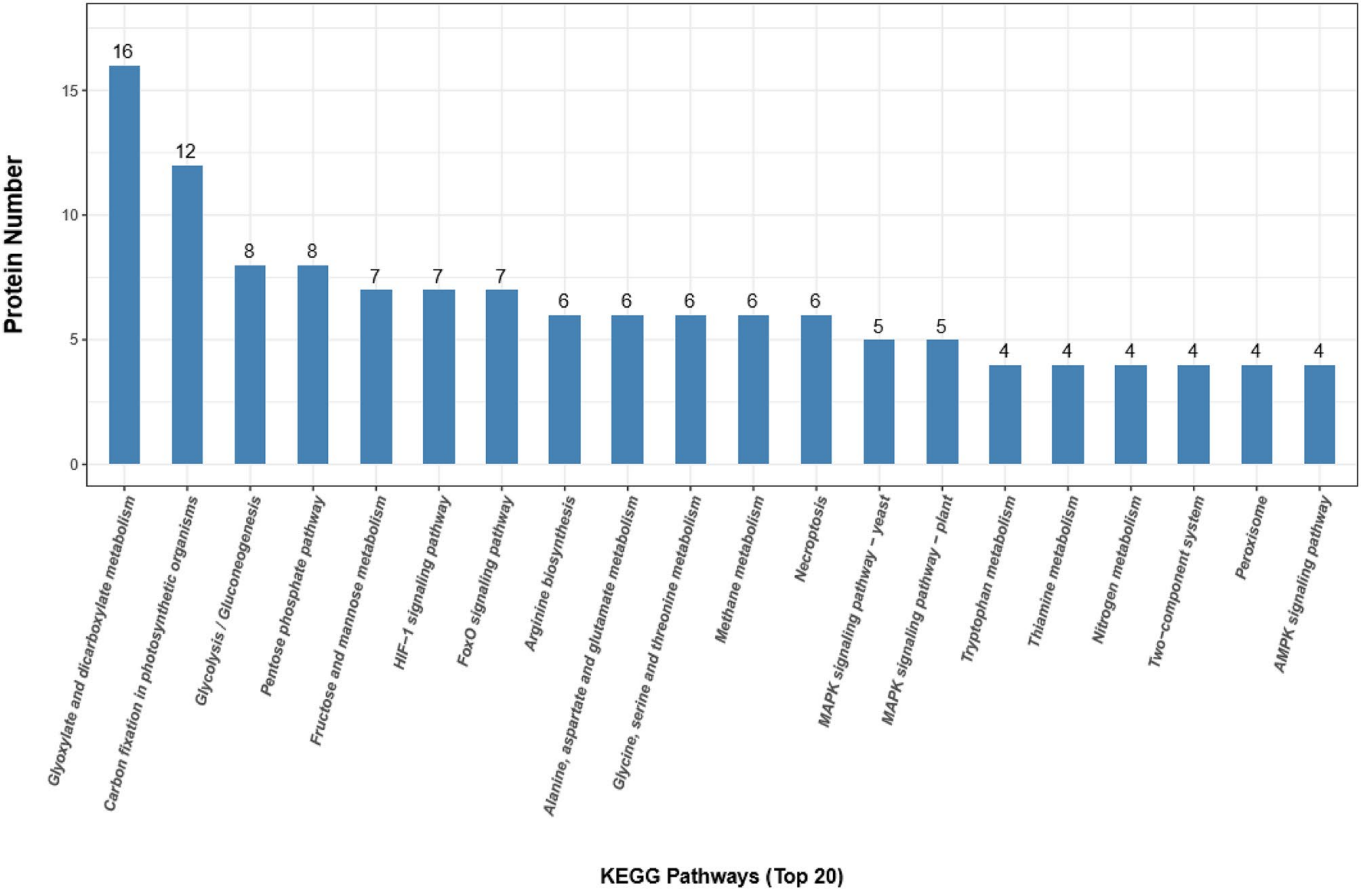
Kyoto Encyclopedia of Genes and Genomes (KEGG) pathway enrichment analyses for the 17K-interacting host genes^14^.

After screening, a total of 183 yeast clones were collected from the SD/-Trp/-Leu/-His/-Ade (QDO) agar plates. In total, 114 cDNA fragments were obtained by colony PCR amplification and sequentially sequenced. We used BLASTn search, gene ontology (GO), and the Kyoto Encyclopedia of Genes and Genome (KEGG) databases to identify the main functional groups of the acquired clones. Based on the results, a total of 90 genes were annotated. The full list of specific annotation results is shown in Supplementary Tables S1–S3. The identified genes were subjected to a GO enrichment analysis. The 90 acquired genes were classified into 35 functional groups, which belonged to three main categories: biological processes (BP), cellular components (CC), and molecular functions (MF). The main subcategories within BP were cellular processes, metabolic processes, and biological regulation. Within the MF category, catalytic activity and binding were predominant. The three main CC subcategories were cell, cell parts, and organelles (Fig. 3). In addition, 49 KEGG pathways for the acquired genes were identified (Table S3). The pathways with the highest numbers of screened genes were glyoxylate and dicarboxylate metabolism, carbon fixation in photosynthetic organisms, and glycolysis/gluconeogenesis (Fig. 4). Based on the analysis, we found that most screened genes were associated with signal transduction (KO04066, KO04068, KO04011, KO04016, KO02020, KO04152, KO04371, KO04020, KO04022, KO04151, KO04014, and KO04150), amino acid metabolism (KO00220, KO00250, KO00260, KO00380, and KO00290), carbohydrate metabolism (KO00630, KO00010, KO00030, KO00051, KO00520, KO00500, KO00562, and KO00040), and energy metabolism (KO00710, KO00680, and KO00910).

#### Genes involved in signal transduction

A subset of genes were related to signal transduction pathways, including the HIF-1 signaling pathway (KO04066), forkhead box O (FOXO) signaling pathway (KP04068), MAPK signaling pathway (KO04011 and KO04016), two-component pathway (KO02020), AMPK signaling pathway (KO04152), Apelin signaling pathway (KO04371), calcium signaling pathway (KO04020), cGMP-PKG signaling pathway (KO04022), PI3K-Akt signaling pathway (KO04151), Ras signaling pathway (KO04014), and mTOR signaling pathway (KO04150). In this study, four genes were associated with the MAPK signaling pathway (XM_016653320.1, XM_016590541.1, NM_001325412.1, and NM_001326196.1) (Table S4). FOXOs are key transcription factors (TFs) that protect cells from various stresses. In *Caenorhabditis elegans*, heat shock factor 1 and FOXO together promote a long life span^15^. In plants, the function of the FOXO signaling pathway remains unclear. In total, six genes were associated with the FOXO signaling pathway (XM_016653320.1, XM_016584004.1, XM_016633060.1, XM_016595156.1, NM_001325412.1, and NM_001326196.1) (Table S4).

#### Genes involved in stress responses

Infection by plant viruses induces the expression of a variety of genes that are usually regulated by TFs^16^. TFs are triggers for gene expression and play important roles throughout the lifetime of plants, especially in plant growth, development, and responses to abiotic and biotic stresses^17^. In this study, we identified four TFs after screening, including TF PosF21 (XM_016587072.1), nuclear TF Y subunit C-1-like (XM_009781056.1), zinc finger protein CONSTANS-LIKE 5-like (XM_016580159.1), and GATA TF 5-like (XM_016591336.1). PosF21 shows all the characteristics of a basic region/leucine zipper motif (bZIP) type of DNA-binding domain^18^. As one of the largest TFs, bZIP TFs play pivotal, life-long roles in plant growth^17^.

#### Genes involved in chloroplast-related functions

Photosynthesis, the most fundamental and complex physiological process in plants, played an important role in viral infection. In this study ten photosynthesis and chloroplast related proteins were identified through screening which are light-harvesting complex I chlorophyll a/b binding protein 3(XM_016643125.1), light-harvesting complex I chlorophyll a/b binding protein 4(XM_016594598.1), light-harvesting complex II chlorophyll a/b binding protein 1(XM_016632344.1), psbP domain-containing protein 5(XM_016648932.1), phosphomethylpyrimidine synthase (XM_016635286.1), glutamine synthetase(XM_016584731.1, XM_016625469.1), protochlorophyllide reductase-like(XM_016616425.1), fructose-bisphosphate aldolase 1(XM_016590854.1, XM_016650131.1), probable 1-deoxy-D-xylulose-5-phosphate synthase(NM_001325496.1), ribulose-phosphate 3-epimerase(XM_016659233.1), glyceraldehyde-3-phosphate dehydrogenase B(XM_016624443.1), PTST(XM_016593025.1), and ribulose bisphosphate carboxylase small chain S41(XM_016613904.1) (Table S5). Among them light-harvesting complex II chlorophyll a/b binding protein, psbP domain-containing protein 5, and glutamine synthetase were previously found to be markedly regulated by BYDV-GAV infection^19^.

### Verification of the interactions between the identified host proteins and BYDV-GAV 17K by yeast two-hybrid assays

**Figure 5:**
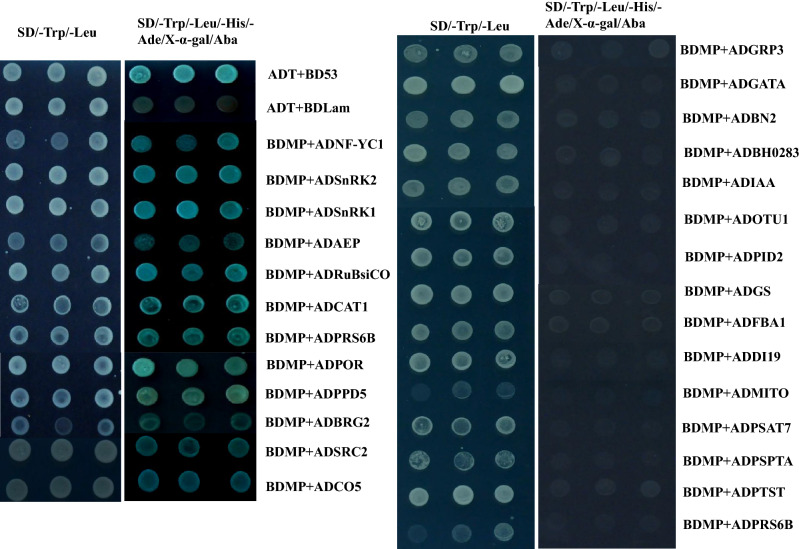
Verification of the interactions between 17K and 27 screened host factors. BD17K was co-transformed with each of 27 host proteins into Y2HGold yeast cells and plated on low-(SD/–Trp/–Leu) and high-(SD/–Trp/–Leu/–His/–Ade/Aba/X-α-Gal) stringency selection media. ADT + BDP53 and ADT + BDLam served as positive and negative controls, respectively.

**Figure 6:**
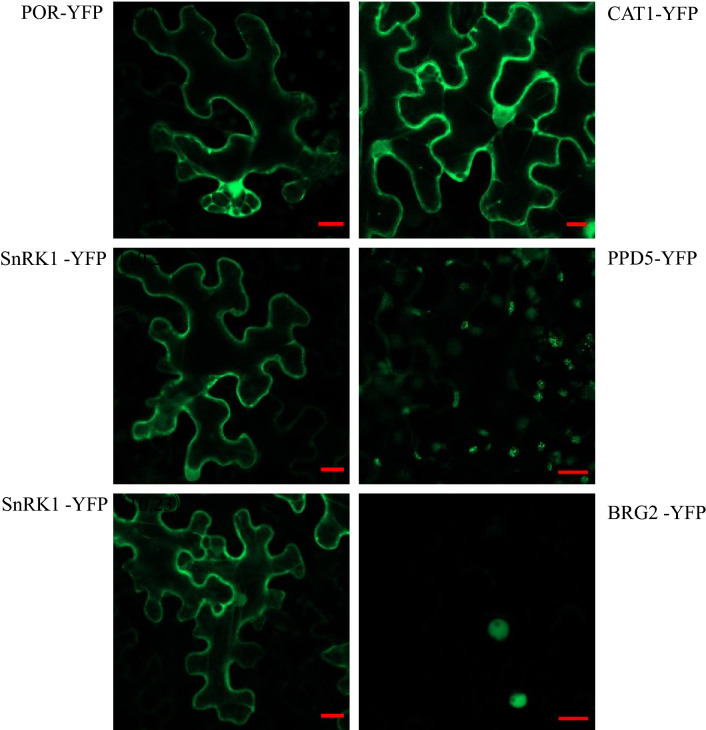
Subcellular localization of six 17K-interacting host proteins in *N. benthamiana* epidermal cells. YFP-tagged POR (POR-YFP), YFP-tagged SnRK1 (SnRK1-YFP), YFP-tagged SnRK2 (SnRK-YFP), YFP-tagged CAT1 (CAT1-YFP), YFP-tagged PPD5 (PPD5-YFP), and YFP-tagged BRG2 (BRG2-YFP) were expressed in N. benthamiana leaves through agrobacterium-mediated transient expression system. Confocal images were taken at 2 dpi using a ZeissLSM710 laser scanning microscope. The bar represents 20 μm.

Based on the screened data, 27 clones involved in different pathways were selected for further research (Tables 1 and S6). We used Y2H method to verify the interactions between BYDV-GAV 17K and the 29 proteins. The cDNAs of the 27 proteins were individually amplified and constructed into the prey vector pGADT7 to generate pGADT7 fusion clones, followed by co-transformation with the pGBKT7-17K into a yeast strain Y2HGold and cultured on SD/-Leu/-Trp (DDO) and SD/-Leu/-Trp/-His/-Ade (QDO) culture media supplemented with X-α-Gal and Aba. Blue colonies were grown from yeast cell co-transformation of BD-17K in 12 of the 27 host proteins on the QDO/X-α-Gal and Aba culture media (Fig. 6). The 12 proteins are shown as follows: SRC2 homolog (SRC2), psbP domain-containing protein 5 (PPD5), catalase isozyme 1-like (CAT1), acidic-endochitinase P (AEP), 26S protease regulatory subunit 6B homolog, sucrose nonfermenting 1 (SNF1)-related protein kinase regulatory subunit beta-2-like (SnRK2), SNF1-related protein kinase regulatory subunit beta-1-like (SnRK1), nuclear TF Y subunit C-1-like (NF-YC1), protochlorophyllide reductase-like (POR), ribulose bisphosphate carboxylase small chain S41 (RuBisCo), zinc finger protein CONSTANS-LIKE 5-like (CO5), and BOI-related E3 ubiquitin-protein ligase 2 (BRG2) . Yeast cells co-transformed in pairs with the other 15 prey vectors, and pGBKT7-17K were able to grow on DDO media but showed no growth on QDO/X-α-Gal and Aba media (Fig. 5).

**Table 1:** The candidate proteins used for yeast two-hybrid analysis.

Accession	Description	Construct	ORF (bp)
Niben101Ctg12075g00002.1	Calcium-dependent lipid-binding (CaLB domain) family protein LENGTH = 352 IPR000008 (C2 domain)	ADSRC2	933
Niben101Scf01371g07014.1	Zinc finger protein 574 IPR013087 (Zinc finger C2H2-type/integrase DNA-binding domain)	ADIDD2	1494
Niben101Scf00777g04013.1	PsbP domain-containing protein 5, chloroplastic IPR016123 (Mog1/PsbP, alpha/beta/alpha sandwich)	ADPPD5	888
Niben101Scf00466g00012.1	Fructose-bisphosphate aldolase 1 LENGTH = 399 IPR000741 (Fructose-bisphosphate aldolase, class-I), IPR013785 (Aldolase-type TIM barrel), IPR029768 (Fructose-bisphosphate aldolase class-I active site)	ADALDP1	1197
Niben101Scf06726g00033.1	Ras-related protein Rab-1D IPR001806 (Small GTPase superfamily), IPR002041 (Ran GTPase), IPR005225 (Small GTP-binding protein domain), IPR027417 (P-loop containing nucleoside triphosphate hydrolase)	ADRABG3	621
Niben101Scf00206g00028.1	IAA-amino acid hydrolase ILR1-like 4 IPR002933 (Peptidase M20)	ADILR1	1326
Niben101Scf07109g00001.1	Proteasome subunit alpha type-4–1 IPR000426 (Proteasome alpha-subunit, N-terminal domain), IPR001353 (Proteasome, subunit alpha/beta), IPR029055 (Nucleophile aminohydrolases, N-terminal)	ADPSMA7	747
Niben101Scf14996g00009.1	Catalase IPR002226 (Catalase haem-binding site), IPR010582 (Catalase immune-responsive domain), IPR011614 (Catalase core domain), IPR018028 (Catalase, mono-functional, haem-containing), IPR020835 (Catalase-like domain), IPR024708 (Catalase active site)	ADCAT1	1479
Niben101Scf02041g00002.1	Chitinase 8 IPR016283 (Glycoside hydrolase, family 19), IPR023346 (Lysozyme-like domain)	ADAEP	762
Niben101Scf01085g02014.1	26S protease regulatory subunit 8 homolog IPR003959 (ATPase, AAA-type, core), IPR005937 (26S proteasome subunit P45), IPR027417 (P-loop containing nucleoside triphosphate hydrolase)	ADPRS6B	1245
Niben101Scf21557g01020.1	Nuclear transcription factor Y subunit C-4 IPR009072 (Histone-fold), IPR027170 (Transcriptional activator NFYC/HAP5 subunit)	ADNF-YC1	693
Niben101Scf08266g00005.1	SNF1 protein kinase subunit beta-2 IPR006828 (Association with the SNF1 complex (ASC) domain), IPR014756 (Immunoglobulin E-set), IPR030070 (SNF1-related protein kinase regulatory subunit beta-2)	ADSnRK2	876
Niben101Scf08721g01033.1	Phenazine biosynthesis PhzC/PhzF protein LENGTH = 313 IPR003719 (Phenazine biosynthesis PhzF protein)	ADBH0283	876
Niben101Scf08921g02023.1	Cathepsin B-like cysteine proteinase 6 IPR013128 (Peptidase C1A)	ADzingipain-1	1026
Niben101Scf01036g03001.1	Protochlorophyllide reductase IPR002347 (Glucose/ribitol dehydrogenase)	ADPOR	1194
Niben101Scf07123g00019.1	SNF1 protein kinase subunit beta-3 IPR006828 (Association with the SNF1 complex (ASC) domain), IPR014756 (Immunoglobulin E-set), IPR030067 (SNF1-related protein kinase regulatory subunit beta-1)	ADSnRK1	879
Niben101Scf01433g05003.1	GATA transcription factor 5 IPR016679 (Transcription factor, GATA, plant)	ADGATA5	1161
Niben101Scf01991g05015.1	Ribulose bisphosphate carboxylase small chain 8B, chloroplastic IPR000894 (Ribulose bisphosphate carboxylase small chain, domain), IPR024680 (Ribulose-1,5-bisphosphate carboxylase small subunit, N-terminal), IPR024681 (Ribulose bisphosphate carboxylase, small chain)	ADRuBisCO	546
Niben101Scf01409g06005.1	Zinc finger protein CONSTANS-LIKE 5 IPR000315 (Zinc finger, B-box), IPR010402 (CCT domain)	ADCO5	1155
Niben101Scf18384g00001.1	Bifunctional nuclease 2 IPR003729 (Bifunctional nuclease domain)	ADBFN2	975
Niben101Scf01858g00003.1	Nucleotide-sugar transporter family protein LENGTH = 350 IPR004853 (Triose-phosphate transporter domain)	ADSPPT	1056
Niben101Scf06996g01006.1	Protein DEHYDRATION-INDUCED 19 homolog 3-like	ADDI19	687
Niben101Scf01084g05013.1	Glycine-rich protein precursor [Nicotiana tabacum] IPR010800 (Glycine rich protein)	ADGRP3	396
Niben101Scf04995g03009.1	S-ribonuclease binding protein 1 LENGTH = 325 IPR013083 (Zinc finger, RING/FYVE/PHD-type), IPR017066 (S-ribonuclease binding protein, SBP1, pollen)	ADBRG2	1014
Niben101Scf00952g03003.1	Glutamine synthetase PR-1 IPR008147 (Glutamine synthetase, beta-Grasp), IPR014746 (Glutamine synthetase/guanido kinase, catalytic domain), IPR027302 (Glutamine synthetase, N-terminal conserved site), IPR027303 (Glutamine synthetase, glycine-rich site)	ADGS	1299
Niben101Scf00069g14019.1	5′-AMP-activated protein kinase subunit beta-1 IPR014756 (Immunoglobulin E-set)	ADPTST	900
Niben101Scf01269g05003.1	Ubiquitin thioesterase OTU1 IPR003323 (Ovarian tumour, otubain)	ADOTU1	627

### Subcellular localization patterns of the identified 17K-interacting host factors

**Figure 7:**
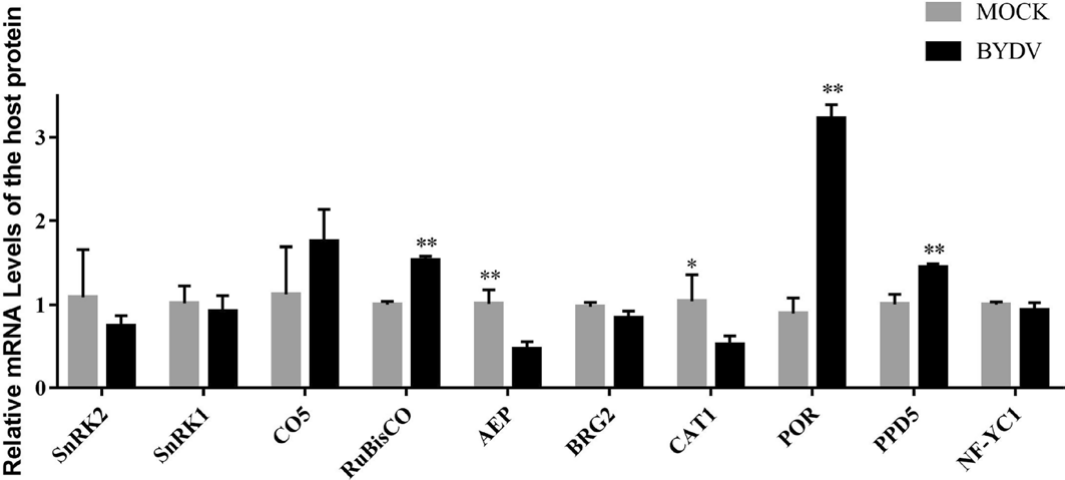
Detection of differentially expressed 17K-interacting host factors under BYDV-GAV infection by qRT-PCR. The relative expression levels of 17K-interacting host factors in mock- and BYDV infected *N. benthamiana* leaves at 5 dpi using qRT-PCR. Data were pooled across experiments and analyzed using *t*-tests. Bars represent the grand means ± standard deviation (SD). *, *P* < 0.05; **, *P* < 0.01.

To determine the subcellular localization of the identified 17K-interacting host factors, we transiently expressed 6 proteins as fusions to the N-terminus of YFP in N. benthamiana leaves. As shown in Fig. 7, POR-YFP and CAT1-YFP showed fluorescence in both the cell periphery and the nucleus. SnRK1-YFP and SnRK2-YFP showed fluorescence in the cell periphery, whereas, BRG2-YFP was perhaps only localized in the nuclear and PPD5 showed the localization at the chloroplast (Figs. 6, S3). The various distributions of identified host proteins suggested 17K protein is possibly involved in several distinct cellular pathways.

### Expression of interacting proteins after BYDV infection

To determine the role of the identified host factors in viral infection, 10 interacting host genes were selected for quantitative RT-PCR (qRT-PCR) analysis using BYDV-inoculated *N. benthamiana* leaves. The results showed that RuBisCo, POR, and PPD5 were significantly up-regulated by viral infection, whereas AEP and CAT1 were significantly down-regulated (Fig. 7). PPD5 was previously reported to be up-regulated in BYDV-infected wheat leaves at 35 dpi, consistent with our results^19^.

## Discussion

Infection by BYDVs causes leaf yellowing and plant dwarfism in wheat, leading to significant economic losses. To realize successful infection, viruses always have to manipulate or utilize many host factors through directly interacting with them. Multifunctional viral proteins such as γb encoded by Barley stripe mosaic virus (BSMV) which are suppressor of RNA silencing and also affects symptom development and seed transmission of BSMV. Previous studies have shown that multiple host factors interact with γb and affects the roles that γb plays during viral infection^20–22^. 17K protein is also a multifunctional protein and its roles in suppression of RNA silencing, virus movement and symptom development are unclear. Identification of interacting host factors will provide insights into the 17 mediated viral process. In this study, we identified 12 host proteins that interact with BYDV 17K. We also tested the possible interaction between 17K and the other BYDV encoded proteins. No interaction was found using Y2H assay.

Reactive oxygen species (ROS) such as hydrogen peroxide (H_2_O_2_) or superoxide anions (O^2^ ^−^) are one of the earliest cellular responses against pathogen infections^20^. Two identified host factors, NbSRC2 and NbCAT1, were involved in ROS production. *Arabidopsis* SRC2 acts as a novel activator of NADPH oxidase AtRbohF-mediated ROS production and may play a role in cold stress^23^. The rapid production of ROS under biotic or abiotic challenges, known as an oxidative response, is an important signal for both local immune responses and cell-to-cell communication^24^. Catalase serves to protect cells from the toxic effects of hydrogen peroxide. The interaction between 17K and NbSRC2/ NbCAT1 may influence viral infection by regulating ROS production.

Two chloroplast proteins were identified: PsbP domain-containing protein 5 and ribulose bisphosphate carboxylase small-chain S41. Plants contain an extensive family of PsbP domain (PPD) proteins, which are localized in the thylakoid lumen. Members of the PsbP family have been shown to exhibit a variety of functions. Except for the function of photosynthetic electron transfer, the *Arabidopsis* ppd5 mutant showed striking morphological defects related to a deficiency in strigolactone biosynthesis^25^. RuBisCo is the most abundant enzyme in plants. It catalyzes the carboxylation of ribulose-1,5-biphosphate in chloroplasts and thus is responsible for fixing CO_2_ during photosynthesis. Hence, 17K may be involved in different pathways in chloroplasts and thus influence symptom development.

TFs are important players in the response to biotic and abiotic stresses. In this study, two TFs were identified: nuclear TF Y subunit C-1-like (NF-YC1) and zinc finger protein CONSTANS-LIKE 5-like (CO5). Nuclear factor Y (NF-Y) is an evolutionarily conserved trimeric TF complex consisting of three subunits: NF-YA, NF-YB, and NF-YC. Recent studies have shown that the NF-Y complex plays multiple essential roles in plant growth, development, and stress responses^26^. Three NF-YC proteins (NF-YC3, NF-YC4, and NF-YC9) are positive regulators of photomorphogenesis^27^. CONSTANS/CONSTANS‐like (CO/COL) belongs to a family of zinc finger TFs and contains one or two B‐box zinc finger regions at the N‐terminus and a CCT (CO, COL, TOC1) domain at the C-terminus^28^. The CO genes have been reported to be involved in many molecular and genetic processes, including photoperiodically regulated developmental processes and abiotic stresses^29–31^. The function of NF-YC1 and CO5 in biotic stress remains unclear. The interaction between 17K and the two TFs may influence the regulation of gene expression by NF-YC1 and CO5, thereby influencing viral accumulation.

Three biotic stress-related genes that interact with 17K were identified, including SNF1-related protein kinase regulatory subunit beta-1-like, SNF1-related protein kinase regulatory subunit beta-2-like, and BOI-related E3 ubiquitin-protein ligase 2.SNF1-related protein kinase 1 (SnRK1) plays a central role in regulating energy and metabolism in plants and has been implicated in responses to abiotic and biotic stresses^32–35^. It is the best-characterized host protein kinase known to be involved in geminivirus infection^34,36,37^. SnRK1 phosphorylation of Rep interferes with viral replication. By contrast, the host defense response is enhanced by SnRK1 phosphorylation of AL2/C2^37^ and β-satellite-encoded βC1 protein^36,38^. Protochlorophyllide reductase is a key enzyme in chlorophyll biosynthesis. BOI-related E3 ubiquitin-protein ligases represent a subclass of RING E3 ligases that contribute to plant disease resistance and abiotic stress tolerance through the suppression of pathogen-induced and stress-induced cell death^39^.

The screening and identification of 17K interacting host factor enhances the understanding of the molecular mechanism of BYDV-GAV infection. Since BYDV has different strains, our study will increase the knowledge on the mechanism of infection by BYDV-GAV and other strains.

## Materials and methods

### Plasmid construction

Primers used for plasmid construction are listed in Table S7. All the available constructs were sequenced.

To construct vectors for yeast two-hybrid analysis, the coding sequence of corresponding genes were amplified and inserted into EcoRI/BamHI digested pGADT7 vectors via homologous recombination.

PVX17K was constructed by introducing 17K into Potato virus X (PVX) vector pGR106 via ClaI and SalI digestion, followed by ligation with T4 DNA ligase (NEB). The fragments used were amplified using primer pairs PVX17KF/PVX17KR.

YFP fusion constructs were constructed using gateway strategy. The corresponding genes were cloned into entry vector pDONR221 (Invitrogen) using primer pairs to generate recombination vector. The resultant clone was used to construct the gateway vector 17K-YFP.

### Yeast two hybrid assays

The *N. tabacum* cDNA library was screened according to the protocol handbook provided by the Matchmaker Gold Yeast Two-Hybrid System (Clontech Laboratories, Mountain View, CA, USA). The full-length BYDV-GAV 17K protein was amplified and cloned into yeast vector pGBKT7 to generate the bait vector BD17K. The cDNA library screening and interaction assay were performed as described previously^40^.

### Plant materials and virus inoculation

*N. benthamiana* plants were grown in pots in a growth room under a 16 h light/8 h dark photoperiod at 25 °C with 60% humidity. For agroinfiltration, Agrobacteria strain GV3101 carrying infectious viral clones were suspended in infiltration buffer (10 mM MgCl2, 10 mM MES, and 200 μM acetosyringone, pH 5.6) at an OD_600_ of 1, kept at room temperature for 2 to 4 h and infiltrated into *N. benthamiana* leaves using a 1-mL needleless syringe.

### Confocal laser scanning microscopy

For the subcellular localization assays, the corresponding constructs were infiltrated into *N. benthamiana* leaves as described previously^41^. The leaves were detached 48 h post-infiltration (hpi) for fluorescence detection. Fluorescence signals were visualized under an inverted spectral confocal laser scanning microscope (LSM 710; Carl Zeiss AG, Oberkochen, Germany). The fluorescence of yellow (YFP) and red (RFP) fluorescent proteins was excited at 514 and 543 nm, respectively.

### Western blotting analysis

Agro-infiltrated leaves were harvested for western blotting assay. Total protein was extracted from 0.2 g leavesusing the extraction buffer containing 20% glycerol, 20 mM Tris–HCl (pH 7.5), 1 mM EDTA, 150 mM NaCl, 1 mM PMSF, 1 × Protease inhibitor cocktail (Sigma, China). Total protein was separated in SDS–polyacrylamide gel electrophoresis, followed by transfer to nitrocellulose membranes. The membranes were probed using anti-PVX CP polyclonal antibodies followed by an HRP-conjugated secondary antibody. The detection signals were developed using an ECL reagent as instructed. PVX CP accumulation were photographed under a chemiluminescence apparatus (Amersham imager 680). CBB staining of the large subunit of RuBisCo served as a loading controls.

### Quantitative RT-PCR

Total RNAs were extracted from harvested *N. benthamiana* protoplasts using Trizol reagent (invitrogen) and treated with RNase-free DNase I. First strand cDNA was synthesized using 1 μg total RNA(Promega). The reaction solution was prepared as follows: an oligo d(T) primer, random primer, and M-MLV reverse transcriptase as instructed. Ten-fold diluted cDNA products were used for qRT-PCR on an Eppendorf Real-Time PCR system using a SYBR Green master mix (Takara). The *Nb-actin* gene were used as internal reference. All the primers used for RT-PCR are listed in Table S7. The relative gene expression levels were calculated using the 2^−△△CT^ method.

### Statement

The study in the manuscript “Identification and functional analyses of host factors interacting with the 17-kDa protein of Barley yellow dwarf virus-GAV” is complied with local and national regulations.

## Supplementary Information

Supplementary Information
