# Supplementary material for: Identification and functional analyses of host factors interacting with the 17-kDa protein of Barley yellow dwarf virus-GAV
Source: Sci Rep. 2021 Apr 19;11:8453. doi: 10.1038/s41598-021-87836-1 (PMC8055683; doi:10.1038/s41598-021-87836-1)
Supplement: Supplementary file 1 — Supplementary Information [file 41598_2021_87836_MOESM1_ESM.pdf]

Identification and functional analyses of host factors interacting with the 17-kDa protein of Barley yellow dwarf virus-GAV

Siyu Chen[1+], Xiaoyu Han[1+], Lingling Yang[1], Qinglun Li[1], Yajuan Shi[1], Honglian Li[1], Linlin Chen[1], Bingjian Sun[1], Yan Shi[1*], Xue Yang[1*]

[1]College of Plant Protection, Henan Agricultural University, Zhengzhou 450002, China; 2732568252@qq.com(S.C.); 15537032218@163.com(X.H.); 1959108900@qq.com(L.Y.); liqinglun96@163.com(Q.L.); 1657568068@qq.com(Y.S.); honglianli@sina.com(H.L.); llchensky@163.com(L.C.); sbj8624@sina.com(B.S.);

*****Correspondence: shiyan00925@126.com(Y.S.); yangxuepphappy@126.com(X.Y.)

[+] These authors contributed equally to this work.

Supplementary Materials

Fig. S1 The original gel and blot of figure 1B

Fig. S2 Determination of the RNA silencing suppressor activity of 17K.. Silencing suppression ability of 17K was tested in GFP-transgenic *N. benthamiana* plants (16c), with transient co-expression of GFP. GFP fluorescence was revealed by UV illumination at 5 dpi with *Agrobacterium* constructs. Three independent experiments were conducted.

Fig. S3 Subcellular localization of YFP-tagged PPD5 (PPD5-YFP) *in planta*. Confocal images were taken at 2 dpi using a ZeissLSM710 laser scanning microscope. Bar represents 20 μm.

Table S1 The full list annotation of the screening results.

Table S2 Gene ontology (GO) analysis of the screened genes. The genes were divided into three categories: cellular component, biological process, and molecular function genes.

Table S3 Kyoto Encyclopedia of Genes and Genomes (KEGG) pathway enrichment analyses.

Table S4 Possible 17K-interacting proteins involved in signal transduction

Table S5 Possible 17K-interacting proteins involved in photosynthesis

Table S6 Classification of the candidate 17K-interacting host proteins

Table S7 Primers used in this paper

Figure S1

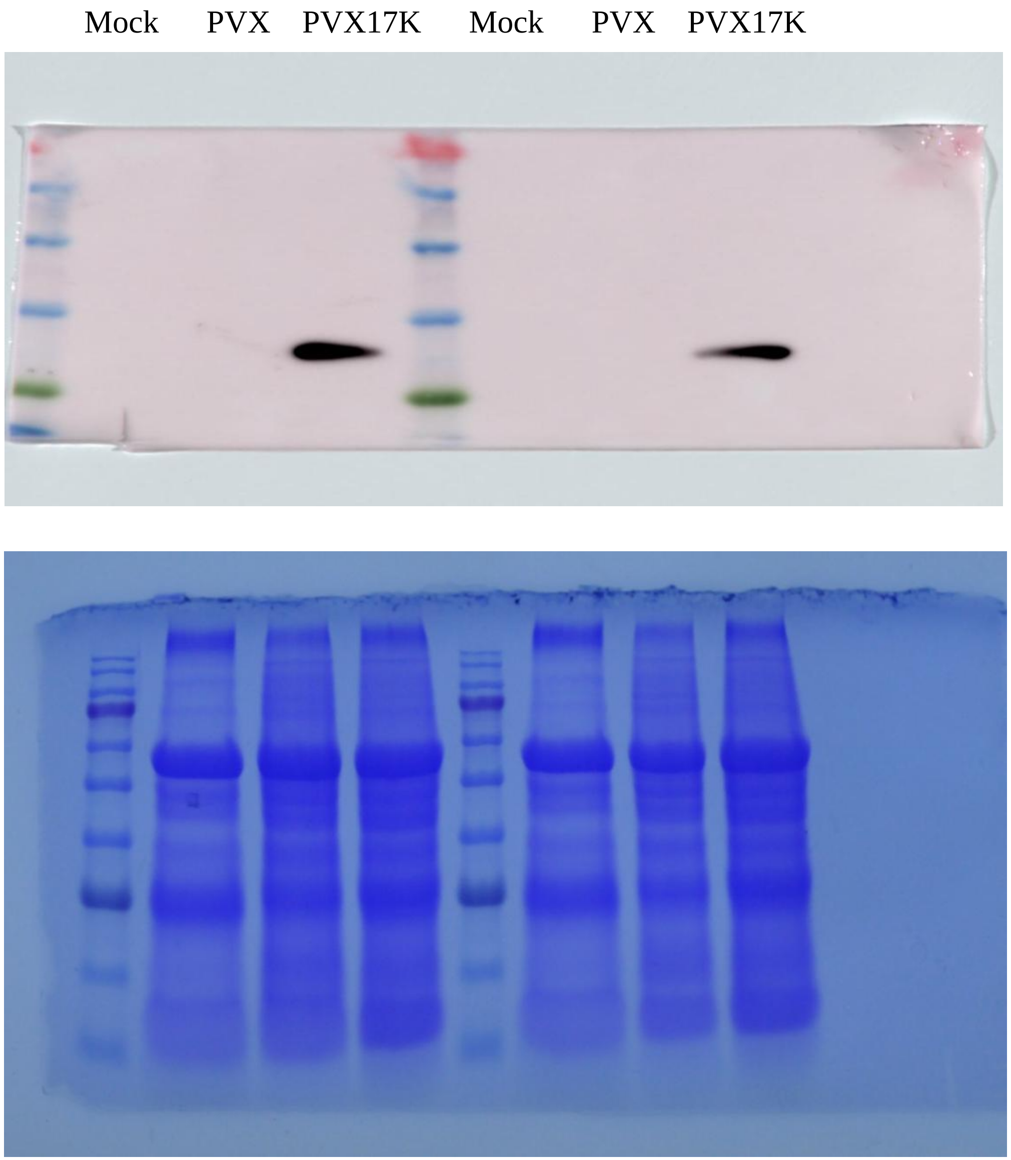

Figure S2

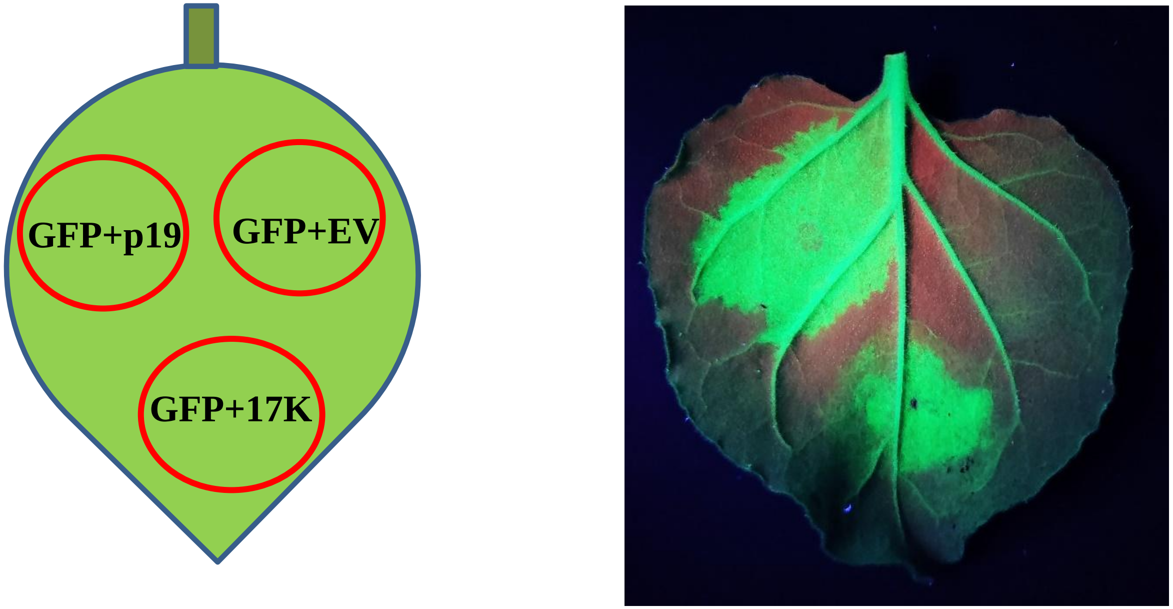

Figure S3

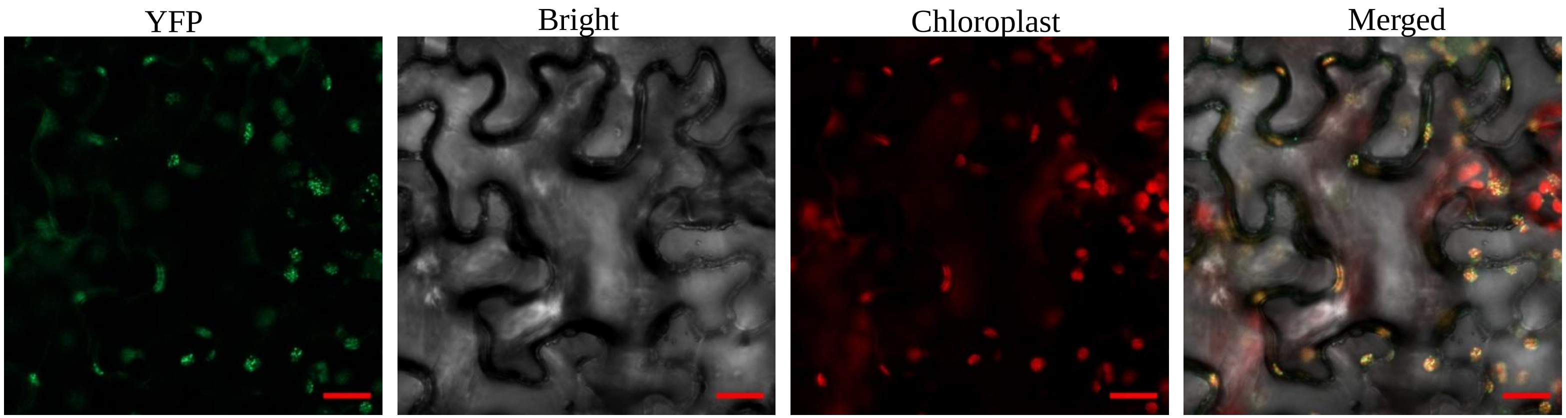

Table S1 screened proteins

	Accession	Description	ORF(bp)	Identity
NO.1	XM_016603969.1	Nicotiana tabacum glycine dehydrogenase (decarboxylating), mitochondrial-like (LOC107783010)	3141	92.46%
NO.2	XM_016596397.1	Nicotiana tabacum pollen-specific leucine-rich repeat extensin-like protein 4	960	81.99%
NO.3	XM_016644389	Nicotiana tabacum keratin, type I cytoskeletal 9-like (LOC107818384), Mrna	468	38.00%
NO.4	XM_016613457.1	Nicotiana tabacum uncharacterized LOC107791394	843	96.58%
NO.5	XM_016606212.1	Nicotiana tabacum argininosuccinate synthase, chloroplastic-like (LOC107784999)	1476	100.00%
NO.6	XM_016639566.1	Nicotiana tabacum far upstream element-binding protein 2-like (LOC107814204)	1968	99.99%
NO.7	XM_016651827.1	Nicotiana tabacum protein SRC2 homolog (LOC107825002)	933	100.00%
NO.8	XM_016628756.1	Nicotiana tabacum actin-97-like (LOC107804820)	1134	96.26%
NO.9	XM_016624436.1	Nicotiana tabacum glucan endo-1,3-beta-glucosidase, basic vacuolar (LOC107801151)	1113	100.00%
NO.10	XM_016577261.1	icotiana tabacum actin-101 (LOC107759349)	1134	98.14%
NO.11	XM_016587072.1	Nicotiana tabacum probable transcription factor PosF21 (LOC107767968)	1773	99.38%
NO.12	XM_016653757.1	Nicotiana tabacum protein indeterminate-domain 2-like (LOC107826732)	1494	40.29%
NO.13	XM_016613884.1	Nicotiana tabacum 2-isopropylmalate synthase B (LOC107791752)	1821	98.35%
NO.14	XM_016629984.1	Nicotiana tabacum eukaryotic translation initiation factor 3 subunit G-like (LOC107805884)	885	92.75%
NO.15	XM_016615771.1	Nicotiana tabacum dnaJ protein homolog (LOC107793424)	1263	97.85%
NO.16	XM_016648932.1	Nicotiana tabacum psbP domain-containing protein 5, chloroplastic-like (LOC107822395)	888	100.00%
NO.17	XM_016644424.1	Nicotiana tabacum mitochondrial outer membrane protein porin of 36 kDa-like	831	100.00%
NO.18	XM_016646948.1	Nicotiana tabacum guanine nucleotide-binding protein subunit beta-2-like (LOC107820634)	1143	96.68%
NO.19	XM_016625469.1	Nicotiana tabacum glutamine synthetase, chloroplastic-like (LOC107802035)	1299	97.18%
NO.20	XM_016635286.1	Nicotiana tabacum phosphomethylpyrimidine synthase, chloroplastic (LOC107810498)	1941	99.64%

NO.21	XM_016650131.1	Nicotiana tabacum fructose-bisphosphate aldolase 1, chloroplastic (LOC107823486)	1188	97.28%
NO.22	XM_009622703.2	Nicotiana tomentosiformis glycine dehydrogenase (decarboxylating), mitochondrial (LOC104112706)	3132	99.80%
NO.23	XM_016590854.1	Nicotiana tabacum fructose-bisphosphate aldolase 1, chloroplastic-like (LOC107771489)	1197	100.00%
NO.24	XM_016656249.1	Nicotiana tabacum ras-related protein RABG3f-like (LOC107828863)	621	100.00%
NO.25	XM_016646129.1	Nicotiana tabacum IAA-amino acid hydrolase ILR1-like 4 (LOC107819938)	1326	99.63%
NO.26	XM_016622070.1	Nicotiana tabacum BEL1-like homeodomain protein 4 (LOC107799012)	2316	97.29%
NO.27	XM_016627134.1	Nicotiana tabacum proteasome subunit alpha type-7-like (LOC107803419)	747	100.00%
NO.28	XM_016653320.1	Nicotiana tabacum catalase isozyme 1-like (LOC107826360)	1479	100.00%
NO.29	XM_016655379.1	Nicotiana tabacum glyceraldehyde-3-phosphate dehydrogenase, cytosolic (LOC107828122)	1023	96.57%
NO.30	XM_016586690.1	Nicotiana tabacum uncharacterized LOC107767630 (LOC107767630)	5424	100.00%
NO.31	XM_016643125.1	Nicotiana tabacum chlorophyll a-b binding protein 8, chloroplastic-like (LOC107817323)	822	99.63%
NO.32	XM_016590541.1	Nicotiana tabacum acidic endochitinase P (LOC107771200)	762	99.72%
NO.33	XM_016625642.1	Nicotiana tabacum actin-related protein 6-like (LOC107802188)	1299	100.00%
NO.34	XM_016635365.1	Nicotiana tabacum importin subunit alpha-2-like (LOC107810574)	1590	99.12%
NO.35	XM_016634045.1	Nicotiana tabacum 26S protease regulatory subunit 6B homolog (LOC107809414)	1245	100.00%
NO.36	XM_016623112.1	Nicotiana tabacum uncharacterized LOC107799967 (LOC107799967)	822	99.38%
NO.37	XM_016655575.1	Nicotiana tabacum nuclear transcription factor Y subunit C-1-like (LOC107828294)	693	100.00%
NO.38	XM_016584004.1	Nicotiana tabacum SNF1-related protein kinase regulatory subunit beta-2-like (LOC107765364)	876	100.00%
NO.39	XM_016591896.1	Nicotiana tabacum uncharacterized isomerase BH0283-like (LOC107772395)	876	99.85%
NO.40	XM_016651825.1	Nicotiana tabacum zingipain-1-like (LOC107824997)	1026	99.96%
NO.41	XM_016633791.1	Nicotiana tabacum NADH--cytochrome b5 reductase 1-like (LOC107809197)	837	100.00%
NO.42	XM_016633060.1	Nicotiana tabacum SNF1-related protein kinase catalytic subunit alpha KIN10-like (LOC107808522)	1539	100.00%

NO.43	XM_016584731.1	Nicotiana tabacum glutamine synthetase, chloroplastic (LOC107766022)	1299	100.00%
NO.44	XM_016616425.1	Nicotiana tabacum protochlorophyllide reductase-like (LOC107793976)	1194	98.97%
NO.45	NM_001325496.1	Nicotiana tabacum probable 1-deoxy-D-xylulose-5-phosphate synthase, chloroplastic (LOC107790348)	2154	100.00%
NO.46	XM_016595156.1	Nicotiana tabacum SNF1-related protein kinase regulatory subunit beta-1-like (LOC107775402)	879	100.00%
NO.47	XM_016585999.1	Nicotiana tabacum uncharacterized LOC107767086 (LOC107767086)	2166	98.75%
NO.48	XM_016655866.1	Nicotiana tabacum BEL1-like homeodomain protein 1 (LOC107828538)	1935	100.00%
NO.49	XM_016595158.1	Nicotiana tabacum cysteine proteinase 3-like (LOC107775429)	1083	100.00%
NO.50	XM_016640620.1	Nicotiana tabacum GTP-binding protein TypA/BipA homolog (LOC107815102)	2040	100.00%
NO.51	XM_016591336.1	Nicotiana tabacum GATA transcription factor 5-like (LOC107771875)	1161	98.54%
NO.52	XM_016621800.1	Nicotiana tabacum ribulose bisphosphate carboxylase/oxygenase activase 2, chloroplastic-like (LOC107798764)	1320	99.76%
NO.53	XM_016659233.1	Nicotiana tabacum ribulose-phosphate 3-epimerase, chloroplastic (LOC107831470)	846	98.19%
NO.54	XM_016635058.1	Nicotiana tabacum polyubiquitin (LOC107810300)	1146	98.31%
NO.55	XM_016650300.1	Nicotiana tabacum protein SRC2-like (LOC107823610)	924	99.53%
NO.56	XM_016632917.1	Nicotiana tabacum probable transcription factor PosF21 (LOC107808400)	1218	99.79%
NO.57	XM_016613904.1	Nicotiana tabacum ribulose bisphosphate carboxylase small chain S41, chloroplastic-like (LOC107791770)	546	100.00%
NO.58	XM_016580159.1	Nicotiana tabacum zinc finger protein CONSTANS-LIKE 5-like (LOC107761869)	1155	98.85%
NO.59	XM_016612689.1	Nicotiana tabacum bifunctional nuclease 2-like (LOC107790732)	975	100.00%
NO.60	XM_016585586.1	Nicotiana tabacum probable sugar phosphate/phosphate translocator At2g25520 (LOC107766750)	1056	98.04%
NO.61	XM_016594598.1	Nicotiana tabacum chlorophyll a-b binding protein P4, chloroplastic-like (LOC107774933)	756	100.00%
NO.62	NM_001326214.1	Nicotiana tabacum cyprosin-like (LOC107828978)	1527	100.00%
NO.63	XM_016632344.1	Nicotiana tabacum chlorophyll a-b binding protein 21, chloroplastic-like (LOC107807891)	798	99.62%
NO.64	XM_016653386.1	Nicotiana tabacum mitochondrial outer membrane protein porin of 34 kDa-like (LOC107826411)	831	100.00%

NO.65	XM_016652740.1	Nicotiana tabacum glucose-6-phosphate isomerase, cytosolic-like (LOC107825825)	1707	100.00%
NO.66	XM_016584562.1	Nicotiana tabacum NAC domain-containing protein 72-like (LOC107765862)	1059	99.86%
NO.67	XM_016587976.1	Nicotiana tabacum protein DEHYDRATION-INDUCED 19 homolog 3-like (LOC107768822)	687	95.63%
NO.68	XM_016602242.1	Nicotiana tabacum glycine-rich protein 3-like (LOC107781526)	396	51.86%
NO.69	XM_016652246.1	Nicotiana tabacum dnaJ protein homolog (LOC107825388)	1254	100.00%
NO.70	NM_001325412.1	Nicotiana tabacum catalase isozyme 1 (LOC107786140)	1479	100.00%
NO.71	XM_016601634.1	Nicotiana tabacum glycine dehydrogenase (decarboxylating), mitochondrial (LOC107781010)	3132	97.92%
NO.72	XM_016630089.1	Nicotiana tabacum aminomethyltransferase, mitochondrial (LOC107805971)	1221	99.88%
NO.73	XM_016615737.1	Nicotiana tabacum probable BOI-related E3 ubiquitin-protein ligase 2 (LOC107793394)	1014	99.88%
NO.74	XM_016626656.1	Nicotiana tabacum glutamate--glyoxylate aminotransferase 2-like (LOC107803058)	1446	93.54%
NO.75	XM_016628566.1	Nicotiana tabacum cinnamoyl-CoA reductase 1-like (LOC107804645)	987	98.25%
NO.76	XM_016580660.1	Nicotiana tabacum phosphoinositide phosphatase SAC3-like (LOC107762314)	2553	99.11%
NO.77	XM_016642214.1	Nicotiana tabacum protein DEHYDRATION-INDUCED 19 homolog 4-like (LOC107816493)	708	100.00%
NO.78	XM_016621192.1	Nicotiana tabacum uncharacterized LOC107798218 (LOC107798218)	1803	98.74%
NO.79	XM_016613519.1	Nicotiana tabacum THO complex subunit 6-like (LOC107791441)	1104	100.00%
NO.80	XM_016650053.1	Nicotiana tabacum glucan endo-1,3-beta-glucosidase, basic vacuolar (LOC107823411)	1113	100.00%
NO.81	XM_016624443.1	Nicotiana tabacum glyceraldehyde-3-phosphate dehydrogenase B, chloroplastic-like (LOC107801158)	1350	100.00%
NO.82	NM_001326196.1	Nicotiana tabacum catalase isozyme 1-like (LOC107828252)	1479	99.35%
NO.83	XM_016593025.1	Nicotiana tabacum protein PTST, chloroplastic-like (LOC107773624)	900	99.04%
NO.84	XM_016655760.1	Nicotiana tabacum ubiquitin thioesterase OTU1-like (LOC107828450)	627	100.00%
NO.85	XM_016614707.1	Nicotiana tabacum coatomer subunit beta-1 (LOC107792487)	2850	100.00%
NO.86	XM_016607172.1	Nicotiana tabacum uncharacterized LOC107785797 (LOC107785797)	882	99.89%

NO.87	XM_016587436.1	Nicotiana tabacum protein SRC2-like (LOC107768315)	921	99.44%
NO.88	XM_016611885.1	Nicotiana tabacum uncharacterized LOC107789999 (LOC107789999)	378	48.02%
NO.89	XM_016627487.1	Nicotiana tabacum xylose isomerase-like (LOC107803724)	1446	100.00%
NO.90	XM_016590101.1	Nicotiana tabacum elongation factor 2 (LOC107770769)	2532	100.00%

Table S2 GO

GO_ID	Term	Type	Seqs_Num	Sequences
GO:0008150	biological_process	Biological Process	33	XM_016622070.1,XM_016655866.1,XM_016634045.1,XM_016635365.1,XM_016586690.1,XM_016626656.1,XM_016615737.1,XM_016590101.1,XM
GO:0000003	reproduction	Biological Process	1	XM_016635365.1
GO:0008152	metabolic process	Biological Process	27	XM_016622070.1,XM_016655866.1,XM_016634045.1,XM_016586690.1,XM_009622703.2,XM_016601634.1,XM_016603969.1,XM_016652740.1,XM
GO:0002376	immune system process	Biological Process	5	XM_016634045.1,XM_016615737.1,XM_016590101.1,XM_016655866.1,XM_016622070.1
GO:0008283	cell proliferation	Biological Process	1	XM_016590101.1
GO:0009987	cellular process	Biological Process	29	XM_016622070.1,XM_016655866.1,XM_016634045.1,XM_016586690.1,XM_016615737.1,XM_016590101.1,XM_016624443.1,XM_016655379.1,XM
GO:0022414	reproductive process	Biological Process	1	XM_016635365.1
GO:0023052	signaling	Biological Process	4	XM_016634045.1,XM_016615737.1,XM_016623112.1,XM_016633060.1
GO:0032501	multicellular organismal process	Biological Process	9	XM_016634045.1,XM_016590101.1,XM_016655379.1,XM_016580660.1,XM_016577261.1,XM_016655866.1,XM_016622070.1,XM_016635365.1,XM
GO:0032502	developmental process	Biological Process	7	XM_016634045.1,XM_016590101.1,XM_016655379.1,XM_016580660.1,XM_016655866.1,XM_016622070.1,XM_016577261.1
GO:0048518	positive regulation of biological process	Biological Process	10	XM_016634045.1,XM_016615737.1,XM_016633060.1,XM_016577261.1,XM_016623112.1,XM_016622070.1,XM_016635365.1,XM_016655866.1,XM
GO:0048519	negative regulation of biological process	Biological Process	10	XM_016622070.1,XM_016655866.1,XM_016577261.1,XM_016615737.1,XM_016623112.1,XM_016652740.1,XM_016655379.1,XM_016634045.1,XM
GO:0050789	regulation of biological process	Biological Process	17	XM_016622070.1,XM_016655866.1,XM_016634045.1,XM_016615737.1,XM_016655575.1,XM_016586690.1,XM_016577261.1,XM_016623112.1,XM
GO:0050896	response to stimulus	Biological Process	13	XM_016634045.1,XM_016615737.1,XM_016590101.1,XM_016655760.1,XM_016633060.1,XM_016577261.1,XM_016652740.1,XM_016624443.1,XM
GO:0051179	localization	Biological Process	4	XM_016635365.1,XM_016656249.1,XM_016633060.1,XM_016623112.1
GO:0051704	multi-organism process	Biological Process	1	XM_016635365.1
GO:0065007	biological regulation	Biological Process	22	XM_016622070.1,XM_016655866.1,XM_016634045.1,XM_016615737.1,XM_009622703.2,XM_016601634.1,XM_016603969.1,XM_016655575.1,XM
GO:0071840	cellular component organization or biogenesis	Biological Process	3	XM_016656249.1,XM_016577261.1,XM_016634045.1
GO:0032504	multicellular organism reproduction	Biological Process	1	XM_016635365.1
GO:0055114	oxidation-reduction process	Biological Process	3	NM_001325412.1,NM_001326196.1,XM_016653320.1
GO:0006807	nitrogen compound metabolic process	Biological Process	19	XM_016622070.1,XM_016655866.1,XM_016634045.1,XM_016586690.1,XM_009622703.2,XM_016601634.1,XM_016603969.1,XM_016655575.1,XM

GO:0009056	catabolic process	Biological Process	9	XM_009622703.2,XM_016601634.1,XM_016603969.1,XM_016635058.1,XM_016633060.1,XM_016656249.1,XM_016634045.1,XM_016655760.1,XM
GO:0009058	biosynthetic process	Biological Process	11	XM_016622070.1,XM_016655866.1,XM_016655575.1,XM_016586690.1,XM_016590101.1,XM_016633060.1,XM_016655379.1,XM_016652740.1,XM
GO:0009892	negative regulation of metabolic process	Biological Process	5	XM_016622070.1,XM_016655866.1,XM_016655379.1,XM_016623112.1,XM_016634045.1
GO:0009893	positive regulation of metabolic process	Biological Process	8	XM_016633060.1,XM_016577261.1,XM_016615737.1,XM_016634045.1,XM_016622070.1,XM_016635365.1,XM_016655866.1,XM_016590101.1
GO:0019222	regulation of metabolic process	Biological Process	12	XM_016622070.1,XM_016655866.1,XM_016655575.1,XM_016586690.1,XM_016634045.1,XM_016633060.1,XM_016577261.1,XM_016655379.1,XM
GO:0032259	methylation	Biological Process	1	XM_016586690.1
GO:0044237	cellular metabolic process	Biological Process	22	XM_016622070.1,XM_016655866.1,XM_016634045.1,XM_016586690.1,XM_009622703.2,XM_016601634.1,XM_016603969.1,XM_016652740.1,XM
GO:0044238	primary metabolic process	Biological Process	22	XM_016622070.1,XM_016655866.1,XM_016634045.1,XM_016586690.1,XM_009622703.2,XM_016601634.1,XM_016603969.1,XM_016652740.1,XM
GO:0044281	small molecule metabolic process	Biological Process	8	XM_009622703.2,XM_016601634.1,XM_016603969.1,XM_016655379.1,XM_016634045.1,XM_016633060.1,XM_016652740.1,XM_016624443.1
GO:0071704	organic substance metabolic process	Biological Process	23	XM_016622070.1,XM_016655866.1,XM_016634045.1,XM_016586690.1,XM_009622703.2,XM_016601634.1,XM_016603969.1,XM_016652740.1,XM
GO:0002253	activation of immune response	Biological Process	2	XM_016634045.1,XM_016615737.1
GO:0002520	immune system development	Biological Process	3	XM_016590101.1,XM_016655866.1,XM_016622070.1
GO:0002682	regulation of immune system process	Biological Process	2	XM_016634045.1,XM_016615737.1
GO:0002684	positive regulation of immune system process	Biological Process	2	XM_016634045.1,XM_016615737.1
GO:0006955	immune response	Biological Process	2	XM_016634045.1,XM_016615737.1
GO:0019882	antigen processing and presentation	Biological Process	1	XM_016634045.1
GO:0014009	glial cell proliferation	Biological Process	1	XM_016590101.1
GO:0007165	signal transduction	Biological Process	4	XM_016634045.1,XM_016615737.1,XM_016623112.1,XM_016633060.1
GO:0006928	movement of cell or subcellular component	Biological Process	1	XM_016577261.1
GO:0007154	cell communication	Biological Process	4	XM_016634045.1,XM_016615737.1,XM_016623112.1,XM_016633060.1
GO:0008219	cell death	Biological Process	6	XM_016624443.1,XM_016655379.1,XM_016577261.1,XM_016615737.1,XM_016623112.1,XM_016652740.1
GO:0016043	cellular component organization	Biological Process	3	XM_016656249.1,XM_016577261.1,XM_016634045.1
GO:0019725	cellular homeostasis	Biological Process	1	XM_016635058.1

GO:0030029	actin filament-based process	Biological Process	1	XM_016577261.1
GO:0048522	positive regulation of cellular process	Biological Process	8	XM_016633060.1,XM_016615737.1,XM_016623112.1,XM_016634045.1,XM_016622070.1,XM_016635365.1,XM_016655866.1,XM_016590101.1
GO:0048523	negative regulation of cellular process	Biological Process	10	XM_016622070.1,XM_016655866.1,XM_016577261.1,XM_016615737.1,XM_016623112.1,XM_016652740.1,XM_016655379.1,XM_016634045.1,XM
GO:0048869	cellular developmental process	Biological Process	4	XM_016590101.1,XM_016622070.1,XM_016655866.1,XM_016577261.1
GO:0050794	regulation of cellular process	Biological Process	17	XM_016622070.1,XM_016655866.1,XM_016634045.1,XM_016615737.1,XM_016655575.1,XM_016586690.1,XM_016577261.1,XM_016623112.1,XM
GO:0051716	cellular response to stimulus	Biological Process	6	XM_016634045.1,XM_016615737.1,XM_016633060.1,XM_016590101.1,XM_016655760.1,XM_016623112.1
GO:0061919	process utilizing autophagic mechanism	Biological Process	2	XM_016633060.1,XM_016656249.1
GO:0044703	multi-organism reproductive process	Biological Process	1	XM_016635365.1
GO:0048609	multicellular organismal reproductive process	Biological Process	1	XM_016635365.1
GO:0007267	cell-cell signaling	Biological Process	1	XM_016634045.1
GO:0023051	regulation of signaling	Biological Process	4	XM_016623112.1,XM_016634045.1,XM_016615737.1,XM_016633060.1
GO:0023056	positive regulation of signaling	Biological Process	1	XM_016634045.1
GO:0023057	negative regulation of signaling	Biological Process	2	XM_016623112.1,XM_016634045.1
GO:0003008	system process	Biological Process	2	XM_016590101.1,XM_016577261.1
GO:0007275	multicellular organism development	Biological Process	7	XM_016634045.1,XM_016590101.1,XM_016580660.1,XM_016655866.1,XM_016622070.1,XM_016577261.1,XM_016655379.1
GO:0044706	multi-multicellular organism process	Biological Process	1	XM_016635365.1
GO:0050879	multicellular organismal movement	Biological Process	1	XM_016590101.1
GO:0051239	regulation of multicellular organismal process	Biological Process	2	XM_016622070.1,XM_016634045.1
GO:0051241	negative regulation of multicellular organismal process	Biological Process	1	XM_016622070.1
GO:0090130	tissue migration	Biological Process	1	XM_016577261.1
GO:0009653	anatomical structure morphogenesis	Biological Process	2	XM_016577261.1,XM_016634045.1
GO:0007568	aging	Biological Process	1	XM_016590101.1
GO:0048646	anatomical structure formation involved in morphogenesis	Biological Process	1	XM_016577261.1

GO:0048856	anatomical structure development	Biological Process	7	XM_016634045.1,XM_016590101.1,XM_016655379.1,XM_016580660.1,XM_016655866.1,XM_016622070.1,XM_016577261.1
GO:0050793	regulation of developmental process	Biological Process	2	XM_016622070.1,XM_016634045.1
GO:0051093	negative regulation of developmental process	Biological Process	1	XM_016622070.1
GO:0044089	positive regulation of cellular component biogenesis	Biological Process	1	XM_016634045.1
GO:0048584	positive regulation of response to stimulus	Biological Process	2	XM_016634045.1,XM_016615737.1
GO:0048585	negative regulation of response to stimulus	Biological Process	2	XM_016623112.1,XM_016634045.1
GO:0032879	regulation of localization	Biological Process	1	XM_016623112.1
GO:0044087	regulation of cellular component biogenesis	Biological Process	1	XM_016634045.1
GO:0048583	regulation of response to stimulus	Biological Process	4	XM_016634045.1,XM_016615737.1,XM_016623112.1,XM_016633060.1
GO:0006950	response to stress	Biological Process	6	XM_016634045.1,XM_016615737.1,XM_016590101.1,XM_016655760.1,XM_016623112.1,XM_016633060.1
GO:0009605	response to external stimulus	Biological Process	2	XM_016615737.1,XM_016590101.1
GO:0009628	response to abiotic stimulus	Biological Process	1	XM_016633060.1
GO:0009719	response to endogenous stimulus	Biological Process	3	XM_016590101.1,XM_016633060.1,XM_016652740.1
GO:0042221	response to chemical	Biological Process	9	XM_016655760.1,XM_016577261.1,XM_016590101.1,XM_016633060.1,XM_016652740.1,XM_016634045.1,XM_016615737.1,XM_016624443.1,XM
GO:0033036	macromolecule localization	Biological Process	2	XM_016635365.1,XM_016623112.1
GO:0051234	establishment of localization	Biological Process	4	XM_016635365.1,XM_016656249.1,XM_016633060.1,XM_016623112.1
GO:0051641	cellular localization	Biological Process	3	XM_016635365.1,XM_016656249.1,XM_016623112.1
GO:0065008	regulation of biological quality	Biological Process	5	XM_009622703.2,XM_016601634.1,XM_016603969.1,XM_016635058.1,XM_016634045.1
GO:0065009	regulation of molecular function	Biological Process	2	XM_016623112.1,XM_016615737.1
GO:0044085	cellular component biogenesis	Biological Process	3	XM_016577261.1,XM_016634045.1,XM_016656249.1
GO:0034641	cellular nitrogen compound metabolic process	Biological Process	10	XM_016622070.1,XM_016655866.1,XM_016586690.1,XM_016655575.1,XM_016590101.1,XM_016634045.1,XM_016655379.1,XM_016635365.1,XM
GO:0051171	regulation of nitrogen compound metabolic process	Biological Process	10	XM_016622070.1,XM_016655866.1,XM_016655575.1,XM_016586690.1,XM_016634045.1,XM_016655379.1,XM_016623112.1,XM_016615737.1,XM
GO:0051172	negative regulation of nitrogen compound metabolic process	Biological Process	4	XM_016622070.1,XM_016655866.1,XM_016655379.1,XM_016623112.1

GO:0051173	positive regulation of nitrogen compound metabolic process	Biological Process	6	XM_016615737.1,XM_016634045.1,XM_016622070.1,XM_016635365.1,XM_016655866.1,XM_016590101.1
GO:1901564	organonitrogen compound metabolic process	Biological Process	14	XM_016634045.1,XM_009622703.2,XM_016601634.1,XM_016603969.1,XM_016590101.1,XM_016633060.1,XM_016627134.1,XM_016635058.1,XM
GO:0009894	regulation of catabolic process	Biological Process	2	XM_016633060.1,XM_016634045.1
GO:0009896	positive regulation of catabolic process	Biological Process	1	XM_016633060.1
GO:0044248	cellular catabolic process	Biological Process	8	XM_009622703.2,XM_016601634.1,XM_016603969.1,XM_016635058.1,XM_016633060.1,XM_016656249.1,XM_016634045.1,XM_016655760.1
GO:0044282	small molecule catabolic process	Biological Process	3	XM_009622703.2,XM_016601634.1,XM_016603969.1
GO:1901575	organic substance catabolic process	Biological Process	8	XM_009622703.2,XM_016601634.1,XM_016603969.1,XM_016635058.1,XM_016656249.1,XM_016634045.1,XM_016655760.1,XM_016652740.1
GO:0009889	regulation of biosynthetic process	Biological Process	9	XM_016622070.1,XM_016655866.1,XM_016655575.1,XM_016586690.1,XM_016655379.1,XM_016633060.1,XM_016634045.1,XM_016635365.1,XM
GO:0009890	negative regulation of biosynthetic process	Biological Process	3	XM_016622070.1,XM_016655866.1,XM_016655379.1
GO:0009891	positive regulation of biosynthetic process	Biological Process	5	XM_016634045.1,XM_016622070.1,XM_016635365.1,XM_016655866.1,XM_016590101.1
GO:0044249	cellular biosynthetic process	Biological Process	9	XM_016622070.1,XM_016655866.1,XM_016655575.1,XM_016586690.1,XM_016590101.1,XM_016655379.1,XM_016633060.1,XM_016634045.1,XM
GO:0044283	small molecule biosynthetic process	Biological Process	2	XM_016633060.1,XM_016652740.1
GO:1901576	organic substance biosynthetic process	Biological Process	11	XM_016622070.1,XM_016655866.1,XM_016655575.1,XM_016586690.1,XM_016590101.1,XM_016633060.1,XM_016655379.1,XM_016634045.1,XM
GO:0010605	negative regulation of macromolecule metabolic process	Biological Process	5	XM_016622070.1,XM_016655866.1,XM_016655379.1,XM_016623112.1,XM_016634045.1
GO:0031324	negative regulation of cellular metabolic process	Biological Process	4	XM_016622070.1,XM_016655866.1,XM_016655379.1,XM_016623112.1
GO:0010604	positive regulation of macromolecule metabolic process	Biological Process	8	XM_016577261.1,XM_016633060.1,XM_016615737.1,XM_016634045.1,XM_016622070.1,XM_016635365.1,XM_016655866.1,XM_016590101.1
GO:0031325	positive regulation of cellular metabolic process	Biological Process	7	XM_016633060.1,XM_016615737.1,XM_016634045.1,XM_016622070.1,XM_016635365.1,XM_016655866.1,XM_016590101.1
GO:0031323	regulation of cellular metabolic process	Biological Process	11	XM_016622070.1,XM_016655866.1,XM_016655575.1,XM_016586690.1,XM_016634045.1,XM_016633060.1,XM_016655379.1,XM_016623112.1,XM
GO:0060255	regulation of macromolecule metabolic process	Biological Process	12	XM_016622070.1,XM_016655866.1,XM_016655575.1,XM_016586690.1,XM_016577261.1,XM_016633060.1,XM_016655379.1,XM_016623112.1,XM
GO:0062012	regulation of small molecule metabolic process	Biological Process	1	XM_016634045.1
GO:0080090	regulation of primary metabolic process	Biological Process	11	XM_016622070.1,XM_016655866.1,XM_016655575.1,XM_016586690.1,XM_016634045.1,XM_016655379.1,XM_016623112.1,XM_016615737.1,XM
GO:0043414	macromolecule methylation	Biological Process	1	XM_016586690.1
GO:0006081	cellular aldehyde metabolic process	Biological Process	1	XM_016652740.1

GO:0006082	organic acid metabolic process	Biological Process	5	XM_009622703.2,XM_016601634.1,XM_016603969.1,XM_016634045.1,XM_016633060.1
GO:0006725	cellular aromatic compound metabolic process	Biological Process	6	XM_016622070.1,XM_016655866.1,XM_016586690.1,XM_016655575.1,XM_016634045.1,XM_016635365.1
GO:0006793	phosphorus metabolic process	Biological Process	4	XM_016634045.1,XM_016633060.1,XM_016652740.1,XM_016623112.1
GO:0017144	drug metabolic process	Biological Process	3	XM_009622703.2,XM_016601634.1,XM_016603969.1
GO:0042133	neurotransmitter metabolic process	Biological Process	3	XM_009622703.2,XM_016601634.1,XM_016603969.1
GO:0042180	cellular ketone metabolic process	Biological Process	1	XM_016634045.1
GO:0044255	cellular lipid metabolic process	Biological Process	1	XM_016633060.1
GO:0044260	cellular macromolecule metabolic process	Biological Process	14	XM_016622070.1,XM_016655866.1,XM_016634045.1,XM_016586690.1,XM_016655575.1,XM_016590101.1,XM_016633060.1,XM_016635058.1,XM
GO:0046483	heterocycle metabolic process	Biological Process	6	XM_016622070.1,XM_016655866.1,XM_016586690.1,XM_016655575.1,XM_016634045.1,XM_016635365.1
GO:0005975	carbohydrate metabolic process	Biological Process	2	XM_016655379.1,XM_016652740.1
GO:0006139	nucleobase-containing compound metabolic process	Biological Process	6	XM_016622070.1,XM_016655866.1,XM_016586690.1,XM_016655575.1,XM_016634045.1,XM_016635365.1
GO:0006520	cellular amino acid metabolic process	Biological Process	4	XM_009622703.2,XM_016601634.1,XM_016603969.1,XM_016634045.1
GO:0006629	lipid metabolic process	Biological Process	2	XM_016633060.1,XM_016656249.1
GO:0019538	protein metabolic process	Biological Process	11	XM_016634045.1,XM_016590101.1,XM_016633060.1,XM_016627134.1,XM_016635058.1,XM_016655760.1,XM_016655379.1,XM_016623112.1,XM
GO:0005996	monosaccharide metabolic process	Biological Process	1	XM_016655379.1
GO:0019637	organophosphate metabolic process	Biological Process	1	XM_016652740.1
GO:0043170	macromolecule metabolic process	Biological Process	17	XM_016622070.1,XM_016655866.1,XM_016634045.1,XM_016586690.1,XM_016655575.1,XM_016590101.1,XM_016633060.1,XM_016627134.1,XM
GO:1901135	carbohydrate derivative metabolic process	Biological Process	1	XM_016652740.1
GO:1901360	organic cyclic compound metabolic process	Biological Process	7	XM_016622070.1,XM_016655866.1,XM_016586690.1,XM_016655575.1,XM_016633060.1,XM_016634045.1,XM_016635365.1
GO:1901615	organic hydroxy compound metabolic process	Biological Process	1	XM_016633060.1
GO:0002218	activation of innate immune response	Biological Process	2	XM_016634045.1,XM_016615737.1
GO:0002757	immune response-activating signal transduction	Biological Process	2	XM_016634045.1,XM_016615737.1
GO:0048534	hematopoietic or lymphoid organ development	Biological Process	3	XM_016590101.1,XM_016655866.1,XM_016622070.1

GO:0050776	regulation of immune response	Biological Process	2	XM_016634045.1,XM_016615737.1
GO:0050778	positive regulation of immune response	Biological Process	2	XM_016634045.1,XM_016615737.1
GO:0045087	innate immune response	Biological Process	2	XM_016634045.1,XM_016615737.1
GO:0019884	antigen processing and presentation of exogenous antigen	Biological Process	1	XM_016634045.1
GO:0048002	antigen processing and presentation of peptide antigen	Biological Process	1	XM_016634045.1
GO:0002764	immune response-regulating signaling pathway	Biological Process	2	XM_016634045.1,XM_016615737.1
GO:0035556	intracellular signal transduction	Biological Process	4	XM_016634045.1,XM_016615737.1,XM_016623112.1,XM_016633060.1
GO:0007166	cell surface receptor signaling pathway	Biological Process	2	XM_016634045.1,XM_016615737.1
GO:0009966	regulation of signal transduction	Biological Process	4	XM_016623112.1,XM_016634045.1,XM_016615737.1,XM_016633060.1
GO:0009967	positive regulation of signal transduction	Biological Process	1	XM_016634045.1
GO:0009968	negative regulation of signal transduction	Biological Process	2	XM_016623112.1,XM_016634045.1
GO:0023014	signal transduction by protein phosphorylation	Biological Process	2	XM_016634045.1,XM_016623112.1
GO:0030522	intracellular receptor signaling pathway	Biological Process	1	XM_016615737.1
GO:0030048	actin filament-based movement	Biological Process	1	XM_016577261.1
GO:0010646	regulation of cell communication	Biological Process	4	XM_016623112.1,XM_016634045.1,XM_016615737.1,XM_016633060.1
GO:0010647	positive regulation of cell communication	Biological Process	1	XM_016634045.1
GO:0010648	negative regulation of cell communication	Biological Process	2	XM_016623112.1,XM_016634045.1
GO:0012501	programmed cell death	Biological Process	6	XM_016624443.1,XM_016655379.1,XM_016577261.1,XM_016615737.1,XM_016623112.1,XM_016652740.1
GO:0070265	necrotic cell death	Biological Process	1	XM_016615737.1
GO:0010941	regulation of cell death	Biological Process	4	XM_016577261.1,XM_016615737.1,XM_016623112.1,XM_016652740.1
GO:0010942	positive regulation of cell death	Biological Process	1	XM_016623112.1
GO:0060548	negative regulation of cell death	Biological Process	4	XM_016577261.1,XM_016615737.1,XM_016623112.1,XM_016652740.1
GO:0006996	organelle organization	Biological Process	2	XM_016656249.1,XM_016577261.1

GO:0022607	cellular component assembly	Biological Process	3	XM_016577261.1,XM_016634045.1,XM_016656249.1
GO:0032989	cellular component morphogenesis	Biological Process	1	XM_016577261.1
GO:0043933	protein-containing complex subunit organization	Biological Process	1	XM_016634045.1
GO:0051128	regulation of cellular component organization	Biological Process	1	XM_016634045.1
GO:0051130	positive regulation of cellular component organization	Biological Process	1	XM_016634045.1
GO:0097435	supramolecular fiber organization	Biological Process	1	XM_016577261.1
GO:0010992	ubiquitin recycling	Biological Process	1	XM_016635058.1
GO:0030036	actin cytoskeleton organization	Biological Process	1	XM_016577261.1
GO:0045596	negative regulation of cell differentiation	Biological Process	1	XM_016622070.1
GO:0030154	cell differentiation	Biological Process	4	XM_016590101.1,XM_016622070.1,XM_016577261.1,XM_016655866.1
GO:0048468	cell development	Biological Process	1	XM_016577261.1
GO:0045595	regulation of cell differentiation	Biological Process	1	XM_016622070.1
GO:0080135	regulation of cellular response to stress	Biological Process	1	XM_016623112.1
GO:0033554	cellular response to stress	Biological Process	4	XM_016634045.1,XM_016590101.1,XM_016655760.1,XM_016623112.1
GO:0070887	cellular response to chemical stimulus	Biological Process	4	XM_016615737.1,XM_016634045.1,XM_016590101.1,XM_016633060.1
GO:0006914	autophagy	Biological Process	2	XM_016633060.1,XM_016656249.1
GO:0007565	female pregnancy	Biological Process	1	XM_016635365.1
GO:0060135	maternal process involved in female pregnancy	Biological Process	1	XM_016635365.1
GO:0198738	cell-cell signaling by wnt	Biological Process	1	XM_016634045.1
GO:1905114	cell surface receptor signaling pathway involved in cell-cell signaling	Biological Process	1	XM_016634045.1
GO:0003012	muscle system process	Biological Process	2	XM_016590101.1,XM_016577261.1
GO:0003013	circulatory system process	Biological Process	1	XM_016577261.1
GO:0009790	embryo development	Biological Process	1	XM_016634045.1

GO:0048731	system development	Biological Process	6	XM_016590101.1,XM_016580660.1,XM_016655866.1,XM_016622070.1,XM_016577261.1,XM_016634045.1
GO:2000026	regulation of multicellular organismal development	Biological Process	2	XM_016634045.1,XM_016622070.1
GO:0050881	musculoskeletal movement	Biological Process	1	XM_016590101.1
GO:0090131	mesenchyme migration	Biological Process	1	XM_016577261.1
GO:0007164	establishment of tissue polarity	Biological Process	1	XM_016634045.1
GO:0009887	animal organ morphogenesis	Biological Process	2	XM_016577261.1,XM_016634045.1
GO:0022603	regulation of anatomical structure morphogenesis	Biological Process	1	XM_016634045.1
GO:0048729	tissue morphogenesis	Biological Process	2	XM_016577261.1,XM_016634045.1
GO:0010927	cellular component assembly involved in morphogenesis	Biological Process	1	XM_016577261.1
GO:0001824	blastocyst development	Biological Process	1	XM_016634045.1
GO:0009888	tissue development	Biological Process	3	XM_016590101.1,XM_016577261.1,XM_016634045.1
GO:0048513	animal organ development	Biological Process	5	XM_016590101.1,XM_016655866.1,XM_016622070.1,XM_016577261.1,XM_016634045.1
GO:0061061	muscle structure development	Biological Process	2	XM_016577261.1,XM_016590101.1
GO:0031334	positive regulation of protein complex assembly	Biological Process	1	XM_016634045.1
GO:0031349	positive regulation of defense response	Biological Process	2	XM_016634045.1,XM_016615737.1
GO:0032880	regulation of protein localization	Biological Process	1	XM_016623112.1
GO:0051049	regulation of transport	Biological Process	1	XM_016623112.1
GO:0043254	regulation of protein complex assembly	Biological Process	1	XM_016634045.1
GO:0032101	regulation of response to external stimulus	Biological Process	1	XM_016615737.1
GO:0080134	regulation of response to stress	Biological Process	3	XM_016634045.1,XM_016615737.1,XM_016623112.1
GO:0002931	response to ischemia	Biological Process	1	XM_016590101.1
GO:0006952	defense response	Biological Process	2	XM_016634045.1,XM_016615737.1
GO:0006979	response to oxidative stress	Biological Process	1	XM_016590101.1

GO:0035966	response to topologically incorrect protein	Biological Process	1	XM_016655760.1
GO:0009991	response to extracellular stimulus	Biological Process	1	XM_016590101.1
GO:0009725	response to hormone	Biological Process	1	XM_016590101.1
GO:0071495	cellular response to endogenous stimulus	Biological Process	1	XM_016590101.1
GO:1990089	response to nerve growth factor	Biological Process	1	XM_016590101.1
GO:0001101	response to acid chemical	Biological Process	1	XM_016590101.1
GO:0007584	response to nutrient	Biological Process	1	XM_016590101.1
GO:0042493	response to drug	Biological Process	2	XM_016590101.1,XM_016577261.1
GO:0009636	response to toxic substance	Biological Process	2	XM_016590101.1,XM_016577261.1
GO:0010033	response to organic substance	Biological Process	7	XM_016655760.1,XM_016652740.1,XM_016634045.1,XM_016590101.1,XM_016615737.1,XM_016577261.1,XM_016633060.1
GO:0010035	response to inorganic substance	Biological Process	1	XM_016590101.1
GO:0046677	response to antibiotic	Biological Process	2	XM_016590101.1,XM_016577261.1
GO:1901698	response to nitrogen compound	Biological Process	3	XM_016634045.1,XM_016655760.1,XM_016590101.1
GO:1901700	response to oxygen-containing compound	Biological Process	4	XM_016590101.1,XM_016577261.1,XM_016652740.1,XM_016633060.1
GO:0008104	protein localization	Biological Process	2	XM_016635365.1,XM_016623112.1
GO:0070727	cellular macromolecule localization	Biological Process	2	XM_016635365.1,XM_016623112.1
GO:0006810	transport	Biological Process	4	XM_016635365.1,XM_016656249.1,XM_016623112.1,XM_016633060.1
GO:0045184	establishment of protein localization	Biological Process	2	XM_016635365.1,XM_016623112.1
GO:0051649	establishment of localization in cell	Biological Process	2	XM_016635365.1,XM_016656249.1
GO:0046907	intracellular transport	Biological Process	2	XM_016635365.1,XM_016656249.1
GO:0001505	regulation of neurotransmitter levels	Biological Process	3	XM_009622703.2,XM_016601634.1,XM_016603969.1
GO:0042592	homeostatic process	Biological Process	1	XM_016635058.1
GO:0043487	regulation of RNA stability	Biological Process	1	XM_016634045.1

GO:0044092	negative regulation of molecular function	Biological Process	1	XM_016623112.1
GO:0050790	regulation of catalytic activity	Biological Process	2	XM_016623112.1,XM_016615737.1
GO:0043603	cellular amide metabolic process	Biological Process	2	XM_016590101.1,XM_016655379.1
GO:0044106	cellular amine metabolic process	Biological Process	1	XM_016634045.1
GO:0044270	cellular nitrogen compound catabolic process	Biological Process	1	XM_016634045.1
GO:0044271	cellular nitrogen compound biosynthetic process	Biological Process	8	XM_016622070.1,XM_016655866.1,XM_016655575.1,XM_016586690.1,XM_016590101.1,XM_016655379.1,XM_016634045.1,XM_016635365.1
GO:0019219	regulation of nucleobase-containing compound metabolic process	Biological Process	6	XM_016622070.1,XM_016655866.1,XM_016655575.1,XM_016586690.1,XM_016634045.1,XM_016635365.1
GO:0033238	regulation of cellular amine metabolic process	Biological Process	1	XM_016634045.1
GO:0034248	regulation of cellular amide metabolic process	Biological Process	2	XM_016655379.1,XM_016590101.1
GO:0051246	regulation of protein metabolic process	Biological Process	4	XM_016655379.1,XM_016623112.1,XM_016615737.1,XM_016590101.1
GO:0034249	negative regulation of cellular amide metabolic process	Biological Process	1	XM_016655379.1
GO:0045934	negative regulation of nucleobase-containing compound metabolic process	Biological Process	2	XM_016622070.1,XM_016655866.1
GO:0051248	negative regulation of protein metabolic process	Biological Process	2	XM_016655379.1,XM_016623112.1
GO:0045935	positive regulation of nucleobase-containing compound metabolic process	Biological Process	4	XM_016634045.1,XM_016622070.1,XM_016635365.1,XM_016655866.1
GO:0034250	positive regulation of cellular amide metabolic process	Biological Process	1	XM_016590101.1
GO:0051247	positive regulation of protein metabolic process	Biological Process	2	XM_016615737.1,XM_016590101.1
GO:0006518	peptide metabolic process	Biological Process	2	XM_016590101.1,XM_016655379.1
GO:0009308	amine metabolic process	Biological Process	1	XM_016634045.1
GO:1901565	organonitrogen compound catabolic process	Biological Process	6	XM_009622703.2,XM_016601634.1,XM_016603969.1,XM_016635058.1,XM_016634045.1,XM_016655760.1
GO:1901566	organonitrogen compound biosynthetic process	Biological Process	2	XM_016590101.1,XM_016655379.1
GO:0031329	regulation of cellular catabolic process	Biological Process	2	XM_016633060.1,XM_016634045.1
GO:0031331	positive regulation of cellular catabolic process	Biological Process	1	XM_016633060.1
GO:0016054	organic acid catabolic process	Biological Process	3	XM_009622703.2,XM_016601634.1,XM_016603969.1

GO:0019439	aromatic compound catabolic process	Biological Process	1	XM_016634045.1
GO:0042135	neurotransmitter catabolic process	Biological Process	3	XM_009622703.2,XM_016601634.1,XM_016603969.1
GO:0042737	drug catabolic process	Biological Process	3	XM_009622703.2,XM_016601634.1,XM_016603969.1
GO:0044265	cellular macromolecule catabolic process	Biological Process	3	XM_016635058.1,XM_016634045.1,XM_016655760.1
GO:0046700	heterocycle catabolic process	Biological Process	1	XM_016634045.1
GO:0009057	macromolecule catabolic process	Biological Process	3	XM_016635058.1,XM_016634045.1,XM_016655760.1
GO:0016042	lipid catabolic process	Biological Process	1	XM_016656249.1
GO:1901361	organic cyclic compound catabolic process	Biological Process	1	XM_016634045.1
GO:0010556	regulation of macromolecule biosynthetic process	Biological Process	8	XM_016622070.1,XM_016655866.1,XM_016655575.1,XM_016586690.1,XM_016655379.1,XM_016634045.1,XM_016635365.1,XM_016590101.1
GO:0031326	regulation of cellular biosynthetic process	Biological Process	9	XM_016622070.1,XM_016655866.1,XM_016655575.1,XM_016586690.1,XM_016655379.1,XM_016634045.1,XM_016635365.1,XM_016590101.1,XM
GO:0046890	regulation of lipid biosynthetic process	Biological Process	1	XM_016633060.1
GO:0010558	negative regulation of macromolecule biosynthetic process	Biological Process	3	XM_016622070.1,XM_016655866.1,XM_016655379.1
GO:0031327	negative regulation of cellular biosynthetic process	Biological Process	3	XM_016622070.1,XM_016655866.1,XM_016655379.1
GO:0010557	positive regulation of macromolecule biosynthetic process	Biological Process	5	XM_016634045.1,XM_016622070.1,XM_016635365.1,XM_016655866.1,XM_016590101.1
GO:0031328	positive regulation of cellular biosynthetic process	Biological Process	5	XM_016634045.1,XM_016622070.1,XM_016635365.1,XM_016655866.1,XM_016590101.1
GO:0018130	heterocycle biosynthetic process	Biological Process	6	XM_016622070.1,XM_016655866.1,XM_016655575.1,XM_016586690.1,XM_016634045.1,XM_016635365.1
GO:0019438	aromatic compound biosynthetic process	Biological Process	6	XM_016622070.1,XM_016655866.1,XM_016655575.1,XM_016586690.1,XM_016634045.1,XM_016635365.1
GO:0034645	cellular macromolecule biosynthetic process	Biological Process	8	XM_016622070.1,XM_016655866.1,XM_016655575.1,XM_016586690.1,XM_016590101.1,XM_016655379.1,XM_016634045.1,XM_016635365.1
GO:0008610	lipid biosynthetic process	Biological Process	1	XM_016633060.1
GO:0009059	macromolecule biosynthetic process	Biological Process	8	XM_016622070.1,XM_016655866.1,XM_016655575.1,XM_016586690.1,XM_016590101.1,XM_016655379.1,XM_016634045.1,XM_016635365.1
GO:1901362	organic cyclic compound biosynthetic process	Biological Process	7	XM_016622070.1,XM_016655866.1,XM_016655575.1,XM_016586690.1,XM_016633060.1,XM_016634045.1,XM_016635365.1
GO:0010629	negative regulation of gene expression	Biological Process	4	XM_016622070.1,XM_016655866.1,XM_016655379.1,XM_016634045.1
GO:0051253	negative regulation of RNA metabolic process	Biological Process	2	XM_016622070.1,XM_016655866.1

GO:0010563	negative regulation of phosphorus metabolic process	Biological Process	1	XM_016623112.1
GO:0032269	negative regulation of cellular protein metabolic process	Biological Process	2	XM_016655379.1,XM_016623112.1
GO:0010628	positive regulation of gene expression	Biological Process	7	XM_016634045.1,XM_016622070.1,XM_016635365.1,XM_016655866.1,XM_016590101.1,XM_016577261.1,XM_016633060.1
GO:0051254	positive regulation of RNA metabolic process	Biological Process	4	XM_016634045.1,XM_016622070.1,XM_016635365.1,XM_016655866.1
GO:0032270	positive regulation of cellular protein metabolic process	Biological Process	2	XM_016615737.1,XM_016590101.1
GO:0010565	regulation of cellular ketone metabolic process	Biological Process	1	XM_016634045.1
GO:0032268	regulation of cellular protein metabolic process	Biological Process	4	XM_016655379.1,XM_016623112.1,XM_016615737.1,XM_016590101.1
GO:0051174	regulation of phosphorus metabolic process	Biological Process	1	XM_016623112.1
GO:0010468	regulation of gene expression	Biological Process	10	XM_016622070.1,XM_016655866.1,XM_016655575.1,XM_016586690.1,XM_016577261.1,XM_016633060.1,XM_016655379.1,XM_016634045.1,XM
GO:0051252	regulation of RNA metabolic process	Biological Process	6	XM_016622070.1,XM_016655866.1,XM_016655575.1,XM_016586690.1,XM_016634045.1,XM_016635365.1
GO:0006521	regulation of cellular amino acid metabolic process	Biological Process	1	XM_016634045.1
GO:0019216	regulation of lipid metabolic process	Biological Process	1	XM_016633060.1
GO:0001510	RNA methylation	Biological Process	1	XM_016586690.1
GO:0043436	oxoacid metabolic process	Biological Process	5	XM_009622703.2,XM_016601634.1,XM_016603969.1,XM_016634045.1,XM_016633060.1
GO:0006796	phosphate-containing compound metabolic process	Biological Process	4	XM_016634045.1,XM_016633060.1,XM_016623112.1,XM_016652740.1
GO:0006544	glycine metabolic process	Biological Process	3	XM_009622703.2,XM_016601634.1,XM_016603969.1
GO:0006631	fatty acid metabolic process	Biological Process	1	XM_016633060.1
GO:0044267	cellular protein metabolic process	Biological Process	9	XM_016634045.1,XM_016590101.1,XM_016633060.1,XM_016635058.1,XM_016655760.1,XM_016655379.1,XM_016623112.1,XM_016615737.1,XM
GO:0034654	nucleobase-containing compound biosynthetic process	Biological Process	6	XM_016622070.1,XM_016655866.1,XM_016655575.1,XM_016586690.1,XM_016634045.1,XM_016635365.1
GO:0034655	nucleobase-containing compound catabolic process	Biological Process	1	XM_016634045.1
GO:0090304	nucleic acid metabolic process	Biological Process	6	XM_016622070.1,XM_016655866.1,XM_016586690.1,XM_016655575.1,XM_016634045.1,XM_016635365.1
GO:0009063	cellular amino acid catabolic process	Biological Process	3	XM_009622703.2,XM_016601634.1,XM_016603969.1

GO:1901605	alpha-amino acid metabolic process	Biological Process	3	XM_009622703.2,XM_016601634.1,XM_016603969.1
GO:0008202	steroid metabolic process	Biological Process	1	XM_016633060.1
GO:0006508	proteolysis	Biological Process	5	XM_016635058.1,XM_016655760.1,XM_016634045.1,XM_016615737.1,XM_016627134.1
GO:0030163	protein catabolic process	Biological Process	3	XM_016635058.1,XM_016634045.1,XM_016655760.1
GO:0036211	protein modification process	Biological Process	5	XM_016634045.1,XM_016633060.1,XM_016655760.1,XM_016623112.1,XM_016615737.1
GO:0019318	hexose metabolic process	Biological Process	1	XM_016655379.1
GO:0010467	gene expression	Biological Process	10	XM_016622070.1,XM_016655866.1,XM_016655575.1,XM_016586690.1,XM_016590101.1,XM_016577261.1,XM_016633060.1,XM_016655379.1,XM
GO:0043412	macromolecule modification	Biological Process	6	XM_016634045.1,XM_016586690.1,XM_016633060.1,XM_016655760.1,XM_016623112.1,XM_016615737.1
GO:0016125	sterol metabolic process	Biological Process	1	XM_016633060.1
GO:0002758	innate immune response-activating signal transduction	Biological Process	2	XM_016634045.1,XM_016615737.1
GO:0002429	immune response-activating cell surface receptor signaling pathway	Biological Process	1	XM_016634045.1
GO:0030097	hemopoiesis	Biological Process	3	XM_016590101.1,XM_016622070.1,XM_016655866.1
GO:0045088	regulation of innate immune response	Biological Process	2	XM_016634045.1,XM_016615737.1
GO:0045089	positive regulation of innate immune response	Biological Process	2	XM_016634045.1,XM_016615737.1
GO:0002478	antigen processing and presentation of exogenous peptide antigen	Biological Process	1	XM_016634045.1
GO:0002474	antigen processing and presentation of peptide antigen via MHC class I	Biological Process	1	XM_016634045.1
GO:0002768	immune response-regulating cell surface receptor signaling pathway	Biological Process	1	XM_016634045.1
GO:0000165	MAPK cascade	Biological Process	2	XM_016623112.1,XM_016634045.1
GO:0002753	cytoplasmic pattern recognition receptor signaling pathway	Biological Process	1	XM_016615737.1
GO:0007249	I-kappaB kinase/NF-kappaB signaling	Biological Process	1	XM_016615737.1
GO:0031098	stress-activated protein kinase signaling cascade	Biological Process	1	XM_016623112.1
GO:0038061	NIK/NF-kappaB signaling	Biological Process	1	XM_016634045.1
GO:1902531	regulation of intracellular signal transduction	Biological Process	3	XM_016623112.1,XM_016615737.1,XM_016633060.1

GO:1902532	negative regulation of intracellular signal transduction	Biological Process	1	XM_016623112.1
GO:0019221	cytokine-mediated signaling pathway	Biological Process	2	XM_016615737.1,XM_016634045.1
GO:0030111	regulation of Wnt signaling pathway	Biological Process	1	XM_016634045.1
GO:0030177	positive regulation of Wnt signaling pathway	Biological Process	1	XM_016634045.1
GO:0030178	negative regulation of Wnt signaling pathway	Biological Process	1	XM_016634045.1
GO:0070252	actin-mediated cell contraction	Biological Process	1	XM_016577261.1
GO:0006915	apoptotic process	Biological Process	6	XM_016577261.1,XM_016615737.1,XM_016623112.1,XM_016652740.1,XM_016624443.1,XM_016655379.1
GO:0043067	regulation of programmed cell death	Biological Process	4	XM_016577261.1,XM_016615737.1,XM_016623112.1,XM_016652740.1
GO:0043068	positive regulation of programmed cell death	Biological Process	1	XM_016623112.1
GO:0043069	negative regulation of programmed cell death	Biological Process	4	XM_016577261.1,XM_016615737.1,XM_016623112.1,XM_016652740.1
GO:0097300	programmed necrotic cell death	Biological Process	1	XM_016615737.1
GO:0010939	regulation of necrotic cell death	Biological Process	1	XM_016615737.1
GO:0007010	cytoskeleton organization	Biological Process	1	XM_016577261.1
GO:0007033	vacuole organization	Biological Process	1	XM_016656249.1
GO:0070925	organelle assembly	Biological Process	2	XM_016577261.1,XM_016656249.1
GO:0090382	phagosome maturation	Biological Process	1	XM_016656249.1
GO:0065003	protein-containing complex assembly	Biological Process	1	XM_016634045.1
GO:0071824	protein-DNA complex subunit organization	Biological Process	1	XM_016634045.1
GO:0007015	actin filament organization	Biological Process	1	XM_016577261.1
GO:0030239	myofibril assembly	Biological Process	1	XM_016577261.1
GO:0031032	actomyosin structure organization	Biological Process	1	XM_016577261.1
GO:0002244	hematopoietic progenitor cell differentiation	Biological Process	1	XM_016590101.1
GO:0022008	neurogenesis	Biological Process	1	XM_016590101.1

GO:0030099	myeloid cell differentiation	Biological Process	1	XM_016622070.1
GO:0035051	cardiocyte differentiation	Biological Process	1	XM_016577261.1
GO:0035914	skeletal muscle cell differentiation	Biological Process	1	XM_016590101.1
GO:0042692	muscle cell differentiation	Biological Process	1	XM_016577261.1
GO:0055001	muscle cell development	Biological Process	1	XM_016577261.1
GO:0055006	cardiac cell development	Biological Process	1	XM_016577261.1
GO:0070302	regulation of stress-activated protein kinase signaling cascade	Biological Process	1	XM_016623112.1
GO:0034976	response to endoplasmic reticulum stress	Biological Process	3	XM_016634045.1,XM_016655760.1,XM_016590101.1
GO:0071310	cellular response to organic substance	Biological Process	3	XM_016615737.1,XM_016634045.1,XM_016590101.1
GO:0010506	regulation of autophagy	Biological Process	1	XM_016633060.1
GO:0010508	positive regulation of autophagy	Biological Process	1	XM_016633060.1
GO:0016236	macroautophagy	Biological Process	2	XM_016633060.1,XM_016656249.1
GO:0016055	Wnt signaling pathway	Biological Process	1	XM_016634045.1
GO:0006936	muscle contraction	Biological Process	2	XM_016590101.1,XM_016577261.1
GO:0003015	heart process	Biological Process	1	XM_016577261.1
GO:0008015	blood circulation	Biological Process	1	XM_016577261.1
GO:0009792	embryo development ending in birth or egg hatching	Biological Process	1	XM_016634045.1
GO:0007399	nervous system development	Biological Process	2	XM_016590101.1,XM_016580660.1
GO:0072359	circulatory system development	Biological Process	1	XM_016577261.1
GO:2000027	regulation of animal organ morphogenesis	Biological Process	1	XM_016634045.1
GO:0003009	skeletal muscle contraction	Biological Process	1	XM_016590101.1
GO:0001736	establishment of planar polarity	Biological Process	1	XM_016634045.1
GO:0003007	heart morphogenesis	Biological Process	1	XM_016577261.1

GO:0048644	muscle organ morphogenesis	Biological Process	1	XM_016577261.1
GO:0072132	mesenchyme morphogenesis	Biological Process	1	XM_016577261.1
GO:1905330	regulation of morphogenesis of an epithelium	Biological Process	1	XM_016634045.1
GO:0002009	morphogenesis of an epithelium	Biological Process	1	XM_016634045.1
GO:0060415	muscle tissue morphogenesis	Biological Process	1	XM_016577261.1
GO:0060429	epithelium development	Biological Process	1	XM_016634045.1
GO:0060485	mesenchyme development	Biological Process	1	XM_016577261.1
GO:0060537	muscle tissue development	Biological Process	2	XM_016590101.1,XM_016577261.1
GO:0007507	heart development	Biological Process	1	XM_016577261.1
GO:0007517	muscle organ development	Biological Process	2	XM_016590101.1,XM_016577261.1
GO:2000144	positive regulation of DNA-templated transcription, initiation	Biological Process	1	XM_016634045.1
GO:0070201	regulation of establishment of protein localization	Biological Process	1	XM_016623112.1
GO:0090087	regulation of peptide transport	Biological Process	1	XM_016623112.1
GO:2000142	regulation of DNA-templated transcription, initiation	Biological Process	1	XM_016634045.1
GO:0031347	regulation of defense response	Biological Process	2	XM_016634045.1,XM_016615737.1
GO:0000302	response to reactive oxygen species	Biological Process	1	XM_016590101.1
GO:0006986	response to unfolded protein	Biological Process	1	XM_016655760.1
GO:0031667	response to nutrient levels	Biological Process	1	XM_016590101.1
GO:0032355	response to estradiol	Biological Process	1	XM_016590101.1
GO:1990090	cellular response to nerve growth factor stimulus	Biological Process	1	XM_016590101.1
GO:0051593	response to folic acid	Biological Process	1	XM_016590101.1
GO:0033273	response to vitamin	Biological Process	1	XM_016590101.1
GO:0042542	response to hydrogen peroxide	Biological Process	1	XM_016590101.1

GO:0045471	response to ethanol	Biological Process	2	XM_016577261.1,XM_016590101.1
GO:0010243	response to organonitrogen compound	Biological Process	3	XM_016634045.1,XM_016655760.1,XM_016590101.1
GO:0014070	response to organic cyclic compound	Biological Process	2	XM_016590101.1,XM_016652740.1
GO:0033993	response to lipid	Biological Process	2	XM_016590101.1,XM_016652740.1
GO:0034097	response to cytokine	Biological Process	2	XM_016615737.1,XM_016634045.1
GO:0070848	response to growth factor	Biological Process	1	XM_016590101.1
GO:0097305	response to alcohol	Biological Process	2	XM_016577261.1,XM_016590101.1
GO:1901654	response to ketone	Biological Process	1	XM_016652740.1
GO:0034613	cellular protein localization	Biological Process	2	XM_016635365.1,XM_016623112.1
GO:0016192	vesicle-mediated transport	Biological Process	1	XM_016656249.1
GO:0007034	vacuolar transport	Biological Process	1	XM_016656249.1
GO:0071702	organic substance transport	Biological Process	2	XM_016635365.1,XM_016623112.1
GO:0071705	nitrogen compound transport	Biological Process	2	XM_016635365.1,XM_016623112.1
GO:0015031	protein transport	Biological Process	2	XM_016635365.1,XM_016623112.1
GO:0072594	establishment of protein localization to organelle	Biological Process	1	XM_016635365.1
GO:0006886	intracellular protein transport	Biological Process	1	XM_016635365.1
GO:0016197	endosomal transport	Biological Process	1	XM_016656249.1
GO:0016482	cytosolic transport	Biological Process	1	XM_016656249.1
GO:0051169	nuclear transport	Biological Process	1	XM_016635365.1
GO:0043488	regulation of mRNA stability	Biological Process	1	XM_016634045.1
GO:0043086	negative regulation of catalytic activity	Biological Process	1	XM_016623112.1
GO:0051336	regulation of hydrolase activity	Biological Process	1	XM_016615737.1
GO:0051338	regulation of transferase activity	Biological Process	1	XM_016623112.1

GO:0043604	amide biosynthetic process	Biological Process	2	XM_016590101.1,XM_016655379.1
GO:0006417	regulation of translation	Biological Process	2	XM_016655379.1,XM_016590101.1
GO:0030162	regulation of proteolysis	Biological Process	1	XM_016615737.1
GO:0017148	negative regulation of translation	Biological Process	1	XM_016655379.1
GO:0045727	positive regulation of translation	Biological Process	1	XM_016590101.1
GO:0043043	peptide biosynthetic process	Biological Process	2	XM_016590101.1,XM_016655379.1
GO:0061013	regulation of mRNA catabolic process	Biological Process	1	XM_016634045.1
GO:0046395	carboxylic acid catabolic process	Biological Process	3	XM_009622703.2,XM_016601634.1,XM_016603969.1
GO:0006546	glycine catabolic process	Biological Process	3	XM_009622703.2,XM_016601634.1,XM_016603969.1
GO:0006401	RNA catabolic process	Biological Process	1	XM_016634045.1
GO:0043632	modification-dependent macromolecule catabolic process	Biological Process	2	XM_016635058.1,XM_016634045.1
GO:0044257	cellular protein catabolic process	Biological Process	3	XM_016635058.1,XM_016634045.1,XM_016655760.1
GO:2000112	regulation of cellular macromolecule biosynthetic process	Biological Process	8	XM_016622070.1,XM_016655866.1,XM_016655575.1,XM_016586690.1,XM_016655379.1,XM_016634045.1,XM_016635365.1,XM_016590101.1
GO:2001141	regulation of RNA biosynthetic process	Biological Process	6	XM_016622070.1,XM_016655866.1,XM_016655575.1,XM_016586690.1,XM_016634045.1,XM_016635365.1
GO:1902679	negative regulation of RNA biosynthetic process	Biological Process	2	XM_016622070.1,XM_016655866.1
GO:2000113	negative regulation of cellular macromolecule biosynthetic process	Biological Process	3	XM_016622070.1,XM_016655866.1,XM_016655379.1
GO:1902680	positive regulation of RNA biosynthetic process	Biological Process	4	XM_016634045.1,XM_016622070.1,XM_016635365.1,XM_016655866.1
GO:0006351	transcription, DNA-templated	Biological Process	6	XM_016622070.1,XM_016655866.1,XM_016655575.1,XM_016586690.1,XM_016634045.1,XM_016635365.1
GO:0006412	translation	Biological Process	2	XM_016590101.1,XM_016655379.1
GO:0006414	translational elongation	Biological Process	1	XM_016590101.1
GO:0006694	steroid biosynthetic process	Biological Process	1	XM_016633060.1
GO:0032774	RNA biosynthetic process	Biological Process	6	XM_016622070.1,XM_016655866.1,XM_016655575.1,XM_016586690.1,XM_016634045.1,XM_016635365.1
GO:0006402	mRNA catabolic process	Biological Process	1	XM_016634045.1

GO:0045892	negative regulation of transcription, DNA-templated	Biological Process	2	XM_016622070.1,XM_016655866.1
GO:0045936	negative regulation of phosphate metabolic process	Biological Process	1	XM_016623112.1
GO:0031400	negative regulation of protein modification process	Biological Process	1	XM_016623112.1
GO:0045893	positive regulation of transcription, DNA-templated	Biological Process	4	XM_016634045.1,XM_016622070.1,XM_016635365.1,XM_016655866.1
GO:0031401	positive regulation of protein modification process	Biological Process	1	XM_016615737.1
GO:0031399	regulation of protein modification process	Biological Process	2	XM_016623112.1,XM_016615737.1
GO:0019220	regulation of phosphate metabolic process	Biological Process	1	XM_016623112.1
GO:0006355	regulation of transcription, DNA-templated	Biological Process	6	XM_016622070.1,XM_016655866.1,XM_016586690.1,XM_016634045.1,XM_016635365.1,XM_016655575.1
GO:0010608	posttranscriptional regulation of gene expression	Biological Process	3	XM_016655379.1,XM_016634045.1,XM_016590101.1
GO:1903311	regulation of mRNA metabolic process	Biological Process	1	XM_016634045.1
GO:0019752	carboxylic acid metabolic process	Biological Process	5	XM_009622703.2,XM_016601634.1,XM_016603969.1,XM_016634045.1,XM_016633060.1
GO:0016310	phosphorylation	Biological Process	3	XM_016634045.1,XM_016633060.1,XM_016623112.1
GO:0006464	cellular protein modification process	Biological Process	5	XM_016634045.1,XM_016633060.1,XM_016655760.1,XM_016623112.1,XM_016615737.1
GO:0016070	RNA metabolic process	Biological Process	6	XM_016622070.1,XM_016655866.1,XM_016586690.1,XM_016655575.1,XM_016634045.1,XM_016635365.1
GO:1901606	alpha-amino acid catabolic process	Biological Process	3	XM_009622703.2,XM_016601634.1,XM_016603969.1
GO:0009069	serine family amino acid metabolic process	Biological Process	3	XM_009622703.2,XM_016601634.1,XM_016603969.1
GO:0051603	proteolysis involved in cellular protein catabolic process	Biological Process	3	XM_016635058.1,XM_016634045.1,XM_016655760.1
GO:0070646	protein modification by small protein removal	Biological Process	1	XM_016655760.1
GO:0010498	proteasomal protein catabolic process	Biological Process	2	XM_016634045.1,XM_016655760.1
GO:0006006	glucose metabolic process	Biological Process	1	XM_016655379.1
GO:0006396	RNA processing	Biological Process	1	XM_016586690.1
GO:0009451	RNA modification	Biological Process	1	XM_016586690.1
GO:0002220	innate immune response activating cell surface receptor signaling pathway	Biological Process	1	XM_016634045.1

GO:0002221	pattern recognition receptor signaling pathway	Biological Process	1	XM_016615737.1
GO:0050851	antigen receptor-mediated signaling pathway	Biological Process	1	XM_016634045.1
GO:0042590	antigen processing and presentation of exogenous peptide antigen via MHC class I	Biological Process	1	XM_016634045.1
GO:0038093	Fc receptor signaling pathway	Biological Process	1	XM_016634045.1
GO:0043408	regulation of MAPK cascade	Biological Process	1	XM_016623112.1
GO:0043409	negative regulation of MAPK cascade	Biological Process	1	XM_016623112.1
GO:0051403	stress-activated MAPK cascade	Biological Process	1	XM_016623112.1
GO:0070303	negative regulation of stress-activated protein kinase signaling cascade	Biological Process	1	XM_016623112.1
GO:0033209	tumor necrosis factor-mediated signaling pathway	Biological Process	2	XM_016615737.1,XM_016634045.1
GO:0060828	regulation of canonical Wnt signaling pathway	Biological Process	1	XM_016634045.1
GO:0090263	positive regulation of canonical Wnt signaling pathway	Biological Process	1	XM_016634045.1
GO:0090090	negative regulation of canonical Wnt signaling pathway	Biological Process	1	XM_016634045.1
GO:0033275	actin-myosin filament sliding	Biological Process	1	XM_016577261.1
GO:0043066	negative regulation of apoptotic process	Biological Process	4	XM_016577261.1,XM_016615737.1,XM_016623112.1,XM_016652740.1
GO:0042981	regulation of apoptotic process	Biological Process	4	XM_016577261.1,XM_016615737.1,XM_016623112.1,XM_016652740.1
GO:0043065	positive regulation of apoptotic process	Biological Process	1	XM_016623112.1
GO:0060544	regulation of necroptotic process	Biological Process	1	XM_016615737.1
GO:0070266	necroptotic process	Biological Process	1	XM_016615737.1
GO:0034622	cellular protein-containing complex assembly	Biological Process	1	XM_016634045.1
GO:0065004	protein-DNA complex assembly	Biological Process	1	XM_016634045.1
GO:0030240	skeletal muscle thin filament assembly	Biological Process	1	XM_016577261.1
GO:0014866	skeletal myofibril assembly	Biological Process	1	XM_016577261.1
GO:0055003	cardiac myofibril assembly	Biological Process	1	XM_016577261.1

GO:0042063	gliogenesis	Biological Process	1	XM_016590101.1
GO:0055007	cardiac muscle cell differentiation	Biological Process	1	XM_016577261.1
GO:0051146	striated muscle cell differentiation	Biological Process	1	XM_016577261.1
GO:0055002	striated muscle cell development	Biological Process	1	XM_016577261.1
GO:0055013	cardiac muscle cell development	Biological Process	1	XM_016577261.1
GO:0032872	regulation of stress-activated MAPK cascade	Biological Process	1	XM_016623112.1
GO:0036503	ERAD pathway	Biological Process	2	XM_016634045.1,XM_016655760.1
GO:0071345	cellular response to cytokine stimulus	Biological Process	2	XM_016615737.1,XM_016634045.1
GO:0071363	cellular response to growth factor stimulus	Biological Process	1	XM_016590101.1
GO:0035567	non-canonical Wnt signaling pathway	Biological Process	1	XM_016634045.1
GO:0060070	canonical Wnt signaling pathway	Biological Process	1	XM_016634045.1
GO:0006941	striated muscle contraction	Biological Process	2	XM_016590101.1,XM_016577261.1
GO:0030049	muscle filament sliding	Biological Process	1	XM_016577261.1
GO:0060047	heart contraction	Biological Process	1	XM_016577261.1
GO:0043009	chordate embryonic development	Biological Process	1	XM_016634045.1
GO:0090175	regulation of establishment of planar polarity	Biological Process	1	XM_016634045.1
GO:0055008	cardiac muscle tissue morphogenesis	Biological Process	1	XM_016577261.1
GO:0001738	morphogenesis of a polarized epithelium	Biological Process	1	XM_016634045.1
GO:0014706	striated muscle tissue development	Biological Process	2	XM_016590101.1,XM_016577261.1
GO:0048738	cardiac muscle tissue development	Biological Process	1	XM_016577261.1
GO:0060538	skeletal muscle organ development	Biological Process	2	XM_016590101.1,XM_016577261.1
GO:0060261	positive regulation of transcription initiation from RNA polymerase II	Biological Process	1	XM_016634045.1
GO:0051223	regulation of protein transport	Biological Process	1	XM_016623112.1

GO:0060260	regulation of transcription initiation from RNA polymerase II promoter	Biological Process	1	XM_016634045.1
GO:1990416	cellular response to brain-derived neurotrophic factor stimulus	Biological Process	1	XM_016590101.1
GO:0034612	response to tumor necrosis factor	Biological Process	2	XM_016615737.1,XM_016634045.1
GO:0033365	protein localization to organelle	Biological Process	2	XM_016635365.1,XM_016623112.1
GO:0007041	lysosomal transport	Biological Process	1	XM_016656249.1
GO:0015833	peptide transport	Biological Process	2	XM_016635365.1,XM_016623112.1
GO:0042886	amide transport	Biological Process	2	XM_016635365.1,XM_016623112.1
GO:0017038	protein import	Biological Process	1	XM_016635365.1
GO:0006606	protein import into nucleus	Biological Process	1	XM_016635365.1
GO:0006913	nucleocytoplasmic transport	Biological Process	1	XM_016635365.1
GO:0051348	negative regulation of transferase activity	Biological Process	1	XM_016623112.1
GO:0052547	regulation of peptidase activity	Biological Process	1	XM_016615737.1
GO:0043549	regulation of kinase activity	Biological Process	1	XM_016623112.1
GO:2000765	regulation of cytoplasmic translation	Biological Process	1	XM_016590101.1
GO:2000767	positive regulation of cytoplasmic translation	Biological Process	1	XM_016590101.1
GO:0019464	glycine decarboxylation via glycine cleavage system	Biological Process	3	XM_009622703.2,XM_016601634.1,XM_016603969.1
GO:0019941	modification-dependent protein catabolic process	Biological Process	2	XM_016635058.1,XM_016634045.1
GO:1903506	regulation of nucleic acid-templated transcription	Biological Process	6	XM_016622070.1,XM_016655866.1,XM_016655575.1,XM_016586690.1,XM_016634045.1,XM_016635365.1
GO:1903507	negative regulation of nucleic acid-templated transcription	Biological Process	2	XM_016622070.1,XM_016655866.1
GO:1903508	positive regulation of nucleic acid-templated transcription	Biological Process	4	XM_016634045.1,XM_016622070.1,XM_016635365.1,XM_016655866.1
GO:0006366	transcription by RNA polymerase II	Biological Process	5	XM_016622070.1,XM_016655866.1,XM_016586690.1,XM_016634045.1,XM_016635365.1
GO:0006352	DNA-templated transcription, initiation	Biological Process	1	XM_016634045.1
GO:0002181	cytoplasmic translation	Biological Process	1	XM_016590101.1

GO:0097659	nucleic acid-templated transcription	Biological Process	6	XM_016622070.1,XM_016655866.1,XM_016655575.1,XM_016586690.1,XM_016634045.1,XM_016635365.1
GO:0000122	negative regulation of transcription by RNA polymerase II	Biological Process	2	XM_016622070.1,XM_016655866.1
GO:0042326	negative regulation of phosphorylation	Biological Process	1	XM_016623112.1
GO:0001933	negative regulation of protein phosphorylation	Biological Process	1	XM_016623112.1
GO:1903321	negative regulation of protein modification by small protein conjugation or removal	Biological Process	1	XM_016623112.1
GO:0045944	positive regulation of transcription by RNA polymerase II	Biological Process	4	XM_016634045.1,XM_016622070.1,XM_016635365.1,XM_016655866.1
GO:1903322	positive regulation of protein modification by small protein conjugation or removal	Biological Process	1	XM_016615737.1
GO:0001932	regulation of protein phosphorylation	Biological Process	1	XM_016623112.1
GO:1903320	regulation of protein modification by small protein conjugation or removal	Biological Process	2	XM_016623112.1,XM_016615737.1
GO:0042325	regulation of phosphorylation	Biological Process	1	XM_016623112.1
GO:0006357	regulation of transcription by RNA polymerase II	Biological Process	5	XM_016622070.1,XM_016655866.1,XM_016634045.1,XM_016635365.1,XM_016586690.1
GO:0032787	monocarboxylic acid metabolic process	Biological Process	1	XM_016633060.1
GO:0006468	protein phosphorylation	Biological Process	3	XM_016634045.1,XM_016623112.1,XM_016633060.1
GO:0070647	protein modification by small protein conjugation or removal	Biological Process	4	XM_016634045.1,XM_016655760.1,XM_016623112.1,XM_016615737.1
GO:0016071	mRNA metabolic process	Biological Process	1	XM_016634045.1
GO:0009071	serine family amino acid catabolic process	Biological Process	3	XM_009622703.2,XM_016601634.1,XM_016603969.1
GO:0016579	protein deubiquitination	Biological Process	1	XM_016655760.1
GO:0043161	proteasome-mediated ubiquitin-dependent protein catabolic process	Biological Process	1	XM_016634045.1
GO:0002223	stimulatory C-type lectin receptor signaling pathway	Biological Process	1	XM_016634045.1
GO:0002224	toll-like receptor signaling pathway	Biological Process	1	XM_016615737.1
GO:0050852	T cell receptor signaling pathway	Biological Process	1	XM_016634045.1
GO:0002479	antigen processing and presentation of exogenous peptide antigen via MHC class	Biological Process	1	XM_016634045.1
GO:0038095	Fc-epsilon receptor signaling pathway	Biological Process	1	XM_016634045.1

GO:0043405	regulation of MAP kinase activity	Biological Process	1	XM_016623112.1
GO:0032873	negative regulation of stress-activated MAPK cascade	Biological Process	1	XM_016623112.1
GO:0043407	negative regulation of MAP kinase activity	Biological Process	1	XM_016623112.1
GO:0007254	JNK cascade	Biological Process	1	XM_016623112.1
GO:0070897	transcription preinitiation complex assembly	Biological Process	1	XM_016634045.1
GO:0014902	myotube differentiation	Biological Process	1	XM_016577261.1
GO:0014904	myotube cell development	Biological Process	1	XM_016577261.1
GO:0048747	muscle fiber development	Biological Process	1	XM_016577261.1
GO:0046328	regulation of JNK cascade	Biological Process	1	XM_016623112.1
GO:0030433	ubiquitin-dependent ERAD pathway	Biological Process	1	XM_016634045.1
GO:0071356	cellular response to tumor necrosis factor	Biological Process	2	XM_016615737.1,XM_016634045.1
GO:0060071	Wnt signaling pathway, planar cell polarity pathway	Biological Process	1	XM_016634045.1
GO:0060048	cardiac muscle contraction	Biological Process	1	XM_016577261.1
GO:0001701	in utero embryonic development	Biological Process	1	XM_016634045.1
GO:0007519	skeletal muscle tissue development	Biological Process	2	XM_016590101.1,XM_016577261.1
GO:0045899	positive regulation of RNA polymerase II transcriptional preinitiation complex	Biological Process	1	XM_016634045.1
GO:0045898	regulation of RNA polymerase II transcriptional preinitiation complex	Biological Process	1	XM_016634045.1
GO:0034504	protein localization to nucleus	Biological Process	1	XM_016635365.1
GO:0070585	protein localization to mitochondrion	Biological Process	1	XM_016623112.1
GO:0051170	import into nucleus	Biological Process	1	XM_016635365.1
GO:0033673	negative regulation of kinase activity	Biological Process	1	XM_016623112.1
GO:0052548	regulation of endopeptidase activity	Biological Process	1	XM_016615737.1
GO:0045859	regulation of protein kinase activity	Biological Process	1	XM_016623112.1

GO:0006511	ubiquitin-dependent protein catabolic process	Biological Process	2	XM_016634045.1,XM_016635058.1
GO:0006367	transcription initiation from RNA polymerase II promoter	Biological Process	1	XM_016634045.1
GO:0006469	negative regulation of protein kinase activity	Biological Process	1	XM_016623112.1
GO:0031397	negative regulation of protein ubiquitination	Biological Process	1	XM_016623112.1
GO:0031398	positive regulation of protein ubiquitination	Biological Process	1	XM_016615737.1
GO:0031396	regulation of protein ubiquitination	Biological Process	2	XM_016623112.1,XM_016615737.1
GO:0032446	protein modification by small protein conjugation	Biological Process	3	XM_016634045.1,XM_016623112.1,XM_016615737.1
GO:0031145	anaphase-promoting complex-dependent catabolic process	Biological Process	1	XM_016634045.1
GO:0043506	regulation of JUN kinase activity	Biological Process	1	XM_016623112.1
GO:0046329	negative regulation of JNK cascade	Biological Process	1	XM_016623112.1
GO:0043508	negative regulation of JUN kinase activity	Biological Process	1	XM_016623112.1
GO:0051123	RNA polymerase II preinitiation complex assembly	Biological Process	1	XM_016634045.1
GO:0048741	skeletal muscle fiber development	Biological Process	1	XM_016577261.1
GO:2000116	regulation of cysteine-type endopeptidase activity	Biological Process	1	XM_016615737.1
GO:0071900	regulation of protein serine/threonine kinase activity	Biological Process	1	XM_016623112.1
GO:0071901	negative regulation of protein serine/threonine kinase activity	Biological Process	1	XM_016623112.1
GO:0016567	protein ubiquitination	Biological Process	3	XM_016634045.1,XM_016623112.1,XM_016615737.1
GO:0000209	protein polyubiquitination	Biological Process	1	XM_016634045.1

Table S3 KEGG

Map_ID	Map_Name	Seqs	Seqs_Num	URL
ko00630	Glyoxylate and dicarboxylate metabolism（Carbohydrate metabolism ）	XM_016603969.1 XM_016625469.1 XM_016601634.1 XM_016653320.1 XM_016584731.1 XM_016584731.1 XM_016613904.1 XM_016613904.1 XM_016603969.1 NM_001325412.1 XM_016601634.1 XM_016630089.1 XM_016584731.1 XM_016626656.1 NM_001326196.1 XM_016653320.1	16	http://www.kegg.jp/kegg-bin/show_pathway?ko00630+K00281+K01915+K00281+K03781+K01915+K01915+K01602+K01602+K00281+K03781+K00281+K00605+K01915+K14272+K03781+K03781
ko00710	Carbon fixation in photosynthetic organisms（Energy metabolism）	XM_016650131.1 XM_016590854.1 XM_016655379.1 XM_016590854.1 XM_016590854.1 XM_016590854.1 XM_016659233.1 XM_016613904.1 XM_016613904.1 XM_016626656.1 XM_016624443.1 XM_016590854.1	12	http://www.kegg.jp/kegg-bin/show_pathway?ko00710+K01623+K01623+K00134+K01623+K01623+K01623+K01783+K01602+K01602+K14272+K05298+K01623
ko00010	Glycolysis / Gluconeogenesis（Carbohydrate metabolism）	XM_016650131.1 XM_016590854.1 XM_016655379.1 XM_016590854.1 XM_016590854.1 XM_016590854.1 XM_016652740.1 XM_016590854.1	8	http://www.kegg.jp/kegg-bin/show_pathway?ko00010+K01623+K01623+K00134+K01623+K01623+K01623+K01810+K01623
ko00030	Pentose phosphate pathway（Carbohydrate metabolism）	XM_016650131.1 XM_016590854.1 XM_016590854.1 XM_016590854.1 XM_016590854.1 XM_016659233.1 XM_016652740.1 XM_016590854.1	8	http://www.kegg.jp/kegg-bin/show_pathway?ko00030+K01623+K01623+K01623+K01623+K01623+K01783+K01810+K01623
ko00051	Fructose and mannose metabolism（Carbohydrate metabolism）	XM_016650131.1 XM_016590854.1 XM_016590854.1 XM_016590854.1 XM_016590854.1 XM_016590854.1 XM_016627487.1	7	http://www.kegg.jp/kegg-bin/show_pathway?ko00051+K01623+K01623+K01623+K01623+K01623+K01623+K01805
ko04066	HIF-1 signaling pathway（Signal transduction）	XM_016650131.1 XM_016590854.1 XM_016655379.1 XM_016590854.1 XM_016590854.1 XM_016590854.1 XM_016590854.1	7	http://www.kegg.jp/kegg-bin/show_pathway?ko04066+K01623+K01623+K00134+K01623+K01623+K01623
ko04068	FoxO signaling pathway（Signal transduction）	XM_016653320.1 XM_016584004.1 XM_016633060.1 XM_016595156.1 NM_001325412.1 NM_001326196.1 XM_016653320.1	7	http://www.kegg.jp/kegg-bin/show_pathway?ko04068+K03781+K07199+K07198+K07199+K03781+K03781+K03781
ko00220	Arginine biosynthesis（Amino acid metabolism）	XM_016606212.1 XM_016625469.1 XM_016584731.1 XM_016584731.1 XM_016584731.1 XM_016626656.1	6	http://www.kegg.jp/kegg-bin/show_pathway?ko00220+K01940+K01915+K01915+K01915+K01915+K14272
ko00250	Alanine, aspartate and glutamate metabolism（Amino acid metabolism）	XM_016606212.1 XM_016625469.1 XM_016584731.1 XM_016584731.1 XM_016584731.1 XM_016626656.1	6	http://www.kegg.jp/kegg-bin/show_pathway?ko00250+K01940+K01915+K01915+K01915+K01915+K14272
ko00260	Glycine, serine and threonine metabolism（Amino acid metabolism）	XM_016603969.1 XM_016601634.1 XM_016603969.1 XM_016601634.1 XM_016630089.1 XM_016626656.1	6	http://www.kegg.jp/kegg-bin/show_pathway?ko00260+K00281+K00281+K00281+K00281+K00605+K14272
ko00680	Methane metabolism（Energy metabolism）	XM_016650131.1 XM_016590854.1 XM_016590854.1 XM_016590854.1 XM_016590854.1 XM_016590854.1	6	http://www.kegg.jp/kegg-bin/show_pathway?ko00680+K01623+K01623+K01623+K01623+K01623+K01623
ko04217	Necroptosis（Cellular Processes）	XM_016644424.1 XM_016625469.1 XM_016584731.1 XM_016584731.1 XM_016653386.1 XM_016584731.1	6	http://www.kegg.jp/kegg-bin/show_pathway?ko04217+K15040+K01915+K01915+K01915+K15040+K01915
ko04011	MAPK signaling pathway - yeast（ Signal transduction）	XM_016646948.1 XM_016653320.1 NM_001325412.1 NM_001326196.1 XM_016653320.1	5	http://www.kegg.jp/kegg-bin/show_pathway?ko04011+K04536+K03781+K03781+K03781+K03781
ko04016	MAPK signaling pathway - plant（ Signal transduction）	XM_016653320.1 XM_016590541.1 NM_001325412.1 NM_001326196.1 XM_016653320.1	5	http://www.kegg.jp/kegg-bin/show_pathway?ko04016+K03781+K20547+K03781+K03781+K03781
ko00380	Tryptophan metabolism（Amino acid metabolism）	XM_016653320.1 NM_001325412.1 NM_001326196.1 XM_016653320.1	4	http://www.kegg.jp/kegg-bin/show_pathway?ko00380+K03781+K03781+K03781+K03781
ko00730	Thiamine metabolism（ Metabolism of cofactors and vitamins）	XM_016635286.1 NM_001325496.1 XM_016635286.1 XM_016635286.1	4	http://www.kegg.jp/kegg-bin/show_pathway?ko00730+K03147+K01662+K03147+K03147

ko00910	Nitrogen metabolism（Energy metabolism）	XM_016625469.1 XM_016584731.1 XM_016584731.1 XM_016584731.1	4	http://www.kegg.jp/kegg-bin/show_pathway?ko00910+K01915+K01915+K01915+K01915
ko02020	Two-component system（Signal transduction）	XM_016625469.1 XM_016584731.1 XM_016584731.1 XM_016584731.1	4	http://www.kegg.jp/kegg-bin/show_pathway?ko02020+K01915+K01915+K01915+K01915
ko04146	Peroxisome（Cellular Processes）	XM_016653320.1 NM_001325412.1 NM_001326196.1 XM_016653320.1	4	http://www.kegg.jp/kegg-bin/show_pathway?ko04146+K03781+K03781+K03781+K03781
ko04152	AMPK signaling pathway（Signal transduction）	XM_016584004.1 XM_016633060.1 XM_016595156.1 XM_016590101.1	4	http://www.kegg.jp/kegg-bin/show_pathway?ko04152+K07199+K07198+K07199+K03234
ko04371	Apelin signaling pathway（Signal transduction）	XM_016646948.1 XM_016584004.1 XM_016633060.1 XM_016595156.1	4	http://www.kegg.jp/kegg-bin/show_pathway?ko04371+K04536+K07199+K07198+K07199
ko00196	Photosynthesis - antenna proteins （Energy metabolism）	XM_016643125.1 XM_016594598.1 XM_016632344.1	3	http://www.kegg.jp/kegg-bin/show_pathway?ko00196+K08909+K08910+K08912
ko00290	Valine, leucine and isoleucine biosynthesis（Amino acid metabolism）	XM_016613884.1 XM_016619060.1 XM_016613884.1	3	http://www.kegg.jp/kegg-bin/show_pathway?ko00290+K01649+K01703+K01649
ko00520	Amino sugar and nucleotide sugar metabolism （Carbohydrate metabolism）	XM_016590541.1 XM_016633791.1 XM_016652740.1	3	http://www.kegg.jp/kegg-bin/show_pathway?ko00520+K20547+K00326+K01810
ko04141	Protein processing in endoplasmic reticulum（Genetic Information Processing）	XM_016615771.1 XM_016652246.1 XM_016655760.1	3	http://www.kegg.jp/kegg-bin/show_pathway?ko04141+K09503+K09503+K13719
ko04530	Tight junction （Cellular Processes）	XM_016584004.1 XM_016633060.1 XM_016595156.1	3	http://www.kegg.jp/kegg-bin/show_pathway?ko04530+K07199+K07198+K07199
ko00040	Pentose and glucuronate interconversions（Carbohydrate metabolism）	XM_016659233.1 XM_016627487.1	2	http://www.kegg.jp/kegg-bin/show_pathway?ko00040+K01783+K01805
ko00620	Pyruvate metabolism （Carbohydrate metabolism）	XM_016613884.1 XM_016613884.1	2	http://www.kegg.jp/kegg-bin/show_pathway?ko00620+K01649+K01649
ko03013	RNA transport （ Genetic Information Processing）	XM_016629984.1 XM_016613519.1	2	http://www.kegg.jp/kegg-bin/show_pathway?ko03013+K03248+K13175
ko03050	Proteasome （Genetic Information Processing）	XM_016627134.1 XM_016634045.1	2	http://www.kegg.jp/kegg-bin/show_pathway?ko03050+K02731+K03063
ko04020	Calcium signaling pathway（Signal transduction）	XM_016644424.1 XM_016653386.1	2	http://www.kegg.jp/kegg-bin/show_pathway?ko04020+K15040+K15040
ko04022	cGMP-PKG signaling pathway （Signal transduction）	XM_016644424.1 XM_016653386.1	2	http://www.kegg.jp/kegg-bin/show_pathway?ko04022+K15040+K15040
ko04140	Autophagy - animal （ Cellular Processes）	XM_016656249.1 XM_016633060.1	2	http://www.kegg.jp/kegg-bin/show_pathway?ko04140+K07897+K07198
ko04151	PI3K-Akt signaling pathway（Signal transduction）	XM_016646948.1 XM_016633060.1	2	http://www.kegg.jp/kegg-bin/show_pathway?ko04151+K04536+K07198
ko04216	Ferroptosis （Cellular Processes）	XM_016644424.1 XM_016653386.1	2	http://www.kegg.jp/kegg-bin/show_pathway?ko04216+K15040+K15040
ko04218	Cellular senescence （Cellular Processes）	XM_016644424.1 XM_016653386.1	2	http://www.kegg.jp/kegg-bin/show_pathway?ko04218+K15040+K15040
ko00500	Starch and sucrose metabolism（Carbohydrate metabolism）	XM_016652740.1	1	http://www.kegg.jp/kegg-bin/show_pathway?ko00500+K01810
ko00562	Inositol phosphate metabolism （Carbohydrate metabolism）	XM_016580660.1	1	http://www.kegg.jp/kegg-bin/show_pathway?ko00562+K22913
ko00660	C5-Branched dibasic acid metabolism （Carbohydrate metabolism）	XM_016619060.1	1	http://www.kegg.jp/kegg-bin/show_pathway?ko00660+K01703
ko00670	One carbon pool by folate （Metabolism）	XM_016630089.1	1	http://www.kegg.jp/kegg-bin/show_pathway?ko00670+K00605
ko00860	Porphyrin and chlorophyll metabolism （Metabolism）	XM_016616425.1	1	http://www.kegg.jp/kegg-bin/show_pathway?ko00860+K00218
ko00900	Terpenoid backbone biosynthesis （Metabolism）	NM_001325496.1	1	http://www.kegg.jp/kegg-bin/show_pathway?ko00900+K01662
ko00966	Glucosinolate biosynthesis （Metabolism）	XM_016619060.1	1	http://www.kegg.jp/kegg-bin/show_pathway?ko00966+K01703
ko04014	Ras signaling pathway （Signal transduction）	XM_016646948.1	1	http://www.kegg.jp/kegg-bin/show_pathway?ko04014+K04536
ko04137	Mitophagy - animal （Mitophagy - animal）	XM_016656249.1	1	http://www.kegg.jp/kegg-bin/show_pathway?ko04137+K07897
ko04138	Autophagy - yeast （ Cellular Processes）	XM_016656249.1	1	http://www.kegg.jp/kegg-bin/show_pathway?ko04138+K07897
ko04144	Endocytosis （Cellular Processes）	XM_016656249.1	1	http://www.kegg.jp/kegg-bin/show_pathway?ko04144+K07897
ko04145	Phagosome （Cellular Processes）	XM_016656249.1	1	http://www.kegg.jp/kegg-bin/show_pathway?ko04145+K07897
ko04150	mTOR signaling pathway （Signal transduction）	XM_016633060.1	1	http://www.kegg.jp/kegg-bin/show_pathway?ko04150+K07198

Table S4 signal transduction

List of genes involved in the Signal transduction

PathWay	Pathway_definition	number_of_seqs	seqs_kos_list
ko04068	FoxO signaling pathway	6	XM_016653320.1 XM_016584004.1 XM_016633060.1 XM_016595156.1 NM_001325412.1 NM_001326196.1
ko04011	MAPK signaling pathway - yeast	4	XM_016646948.1 XM_016653320.1 NM_001325412.1 NM_001326196.1
ko04016	MAPK signaling pathway - plant	4	XM_016653320.1 XM_016590541.1 NM_001325412.1 NM_001326196.1
ko04152	AMPK signaling pathway	4	XM_016584004.1 XM_016633060.1 XM_016595156.1 XM_016590101.1
ko04371	Apelin signaling pathway	4	XM_016646948.1 XM_016584004.1 XM_016633060.1 XM_016595156.1
ko04066	HIF-1 signaling pathway	3	XM_016650131.1 XM_016590854.1 XM_016655379.1
ko04020	Calcium signaling pathway	2	XM_016644424.1 XM_016653386.1
ko04022	cGMP-PKG signaling pathway	2	XM_016644424.1 XM_016653386.1
ko04151	PI3K-Akt signaling pathway	2	XM_016646948.1 XM_016633060.1
ko02020	Two-component system	2	XM_016625469.1 XM_016584731.1
ko04014	Ras signaling pathway	1	XM_016646948.1
ko04150	mTOR signaling pathway	1	XM_016633060.1

Table S5 Genes involved in photosynthesis

PathWay	Pathway_definition	number_of_seqs	seqs_kos_list
ko00630	Carbon fixation in photosynthetic organisms	5	XM_016650131.1 XM_016590854.1 XM_016659233.1 XM_016613904.1 XM_016624443.1
ko00710	Glyoxylate and dicarboxylate metabolism	3	XM_016625469.1 XM_016584731.1 XM_016613904.1
ko00196	Photosynthesis - antenna proteins	3	XM_016643125.1 XM_016594598.1 XM_016632344.1
ko00730	Thiamine metabolism	2	XM_016635286.1 NM_001325496.1
	Porphyrin and chlorophyll metabolism	1	XM_016616425.1
	Undefined	2	XM_016648932.1 XM_016593025.1

Table S6 classification of selected host proteins

Protein Name	Accession Number	Specie and Accession Number of Homologue	Functional Description	Clone Number
**metabolism**				
leuA(2-isopropylmalate synthase [EC:2.3.3.13])	Niben101Scf08892g00008.1	Nicotiana tabacum XM_016613884.1	Valine, leucine and isoleucine biosynthesis；Pyruvate metabolism	2
katE(catalase [EC:1.11.1.6])	Niben101Scf14996g00009.1	Nicotiana tabacum XM_016653320.1	Tryptophan metabolism;Glyoxylate and dicarboxylate metabolism	2
glnA(glutamine synthetase [EC:6.3.1.2])	Niben101Scf00952g03003.1	Nicotiana tabacum XM_016584731.1	Arginine biosynthesis；Alanine, aspartate and glutamate metabolism;Nitrogen metabolism;Glyoxylate and dicarboxylate metabolism	3
ALDO(fructose-bisphosphate aldolase, class I [EC:4.1.2.13])	Niben101Scf00466g00012.1	Nicotiana tabacum XM_016590854.1	Carbon fixation in photosynthetic organisms；Methane metabolism;Glycolysis / Gluconeogenesis;Pentose phosphate pathway;Fructose and mannose metabolism	5
rbcS(ribulose-bisphosphate carboxylase small chain [EC:4.1.1.39])	Niben101Scf01991g05015.1	Nicotiana tabacum XM_016613904.1	Carbon fixation in photosynthetic organisms;Glyoxylate and dicarboxylate metabolism	2
CHIB(basic endochitinase B [EC:3.2.1.14])	Niben101Scf02041g00002.1	Nicotiana tabacum XM_016590541.1	Amino sugar and nucleotide sugar metabolism	1
por(protochlorophyllide reductase [EC:1.3.1.33])	Niben101Scf01036g03001.1	Nicotiana tabacum XM_016616425.1	Porphyrin and chlorophyll metabolism	1
**Signal transduction**				
ALDO(fructose-bisphosphate aldolase, class I [EC:4.1.2.13])	Niben101Scf00466g00012.1	Nicotiana tabacum XM_016590854.1	HIF-1 signaling pathway	5
katE(catalase [EC:1.11.1.6])	Niben101Scf14996g00009.1	Nicotiana tabacum XM_016653320.1	FoxO signaling pathway；MAPK signaling pathway - yeast/plant；	2
CHIB(basic endochitinase B [EC:3.2.1.14])	Niben101Scf02041g00002.1	Nicotiana tabacum XM_016590541.1	MAPK signaling pathway - plant	1
PRKAB(5'-AMP-activated protein kinase, regulatory beta subunit)	Niben101Scf08266g00005.1	Nicotiana tabacum XM_016584004.1	FoxO signaling pathway;AMPK signaling pathway;Apelin signaling pathway	1
PRKAB(5'-AMP-activated protein kinase, regulatory beta subunit)	Niben101Scf07123g00018.1	Nicotiana tabacum XM_016595156.1	FoxO signaling pathway;AMPK signaling pathway;Apelin signaling pathway	1
glnA(glutamine synthetase [EC:6.3.1.2])	Niben101Scf00952g03003.1	Nicotiana tabacum XM_016584731.1	Two-component system	3
**Cellular Processes**				
RAB7A(Ras-related protein Rab-7A)	Niben101Scf06726g00033.1	Nicotiana tabacum XM_016656249.1	Autophagy - animal;Mitophagy - animal;Autophagy - yeast;Endocytosis;Phagosome	1
katE(catalase [EC:1.11.1.6])	Niben101Scf14996g00009.1	Nicotiana tabacum XM_016653320.1	Peroxisome	2
glnA(glutamine synthetase [EC:6.3.1.2])	Niben101Scf00952g03003.1	Nicotiana tabacum XM_016584731.1	Necroptosis	3
**Genetic Information Processing**				
PSMA7(20S proteasome subunit alpha 4 [EC:3.4.25.1])	Niben101Scf07109g00001.1	Nicotiana tabacum XM_016627134.1	Proteasome	1
PSMC4(26S proteasome regulatory subunit T3)	Niben101Scf01085g02014.1	Nicotiana tabacum XM_016634045.1	Proteasome	1
OTU1(ubiquitin thioesterase OTU1 [EC:3.1.2.-])	Niben101Scf01269g05003.1	Nicotiana tabacum XM_016655760.1	Protein processing in endoplasmic reticulum	1
**Cellular community**				
PRKAB(5'-AMP-activated protein kinase, regulatory beta subunit)	Niben101Scf08266g00005.1	Nicotiana tabacum XM_016584004.1	Tight junction	1
PRKAB(5'-AMP-activated protein kinase, regulatory beta subunit)	Niben101Scf07123g00018.1	Nicotiana tabacum XM_016595156.1	Tight junction	1
**Undefined**				
SRC2(protein SRC2 homolog)	Niben101Ctg12075g00002.1	Nicotiana tabacum XM_016651827.1	Undefined	1
IDD2(protein indeterminate-domain 2-like)	Niben101Scf01371g07014.1	Nicotiana tabacum XM_016653757.1	Undefined	1
PPD5(psbP domain-containing protein 5)	Niben101Scf00777g04013.1	Nicotiana tabacum XM_016648932.1	Undefined	1
ILR1(IAA-amino acid hydrolase ILR1-like 4)	Niben101Scf00206g00028.1	Nicotiana tabacum XM_016646129.1	Undefined	1
NF-YC1(nuclear transcription factor Y subunit C-1-like)	Niben101Scf21557g01020.1	Nicotiana tabacum XM_016655575.1	Undefined	1
BH0283(uncharacterized isomerase BH0283-like)	Niben101Scf08721g01033.1	Nicotiana tabacum XM_016591896.1	Undefined	1

zingipain-1(zingipain-1-like)	Niben101Scf08921g02023.1	Nicotiana tabacum XM_016651825.1	Undefined	1
GATA5(GATA transcription factor 5-like)	Niben101Scf01433g05003.1	Nicotiana tabacum XM_016591336.1	Undefined	1
CO5(zinc finger protein CONSTANS-LIKE 5-like)	Niben101Scf01409g06005.1	Nicotiana tabacum XM_016580159.1	Undefined	1
BFN2(bifunctional nuclease 2-like)	Niben101Scf18384g00001.1	Nicotiana tabacum XM_016612689.1	Undefined	1
[illegible]SPPT(probable sugar phosphate/phosphate translocator At2g25520[illegible]	Niben101Scf01858g00003.1	Nicotiana tabacum XM_016585586.1	Undefined	1
DI19(protein DEHYDRATION-INDUCED 19 homolog 3-like)	Niben101Scf06996g01006.1	Nicotiana tabacum XM_016587976.1	Undefined	1
GRP3(glycine-rich protein 3-like)	Niben101Scf01084g05013.1	Nicotiana tabacum XM_016602242.1	Undefined	1
BRG2(probable BOI-related E3 ubiquitin-protein ligase 2)	Niben101Scf04995g03009.1	Nicotiana tabacum XM_016615737.1	Undefined	1
PTST(protein PTST)	Niben101Scf00069g14019.1	Nicotiana tabacum XM_016593025.1	Undefined	1

Table S7 Primers used in the paper

Primers	Sequences (5'-3')
PVXBYDV17KF	AGAGGTCAGCACCAGCTAGCATCGATATGGCCCAAGGAGAGCAAG
PVXBYDV17KR	AACTTAACCGTTCATCGGCGGTCGACTCACCGTGCTCTCCCTGAA
BP17KF	GGGGACAAGTTTGTACAAAAAAGCAGGCTTCATGGCCCAAGGAGAGCAA
BP17KR	GGGGACCACTTTGTACAAGAAAGCTGGGTCCCGTGCTCTCCCTGAATTC
BD17KF	AATCGACATATGATGGCCCAAGGAGAGCAAG
BD17KR	TCCGGATCCTCACCGTGCTCTCCCTGAA
ADP1F	ATATGGCCATGGAGGCCAGTGAATTCATGTTCTTTGAAATTCTTA
ADP1R	ATCTGCAGCTCGAGCTCGATGGATCCCTAAAAACCCACAGAGTCA
ADP2F	ATATGGCCATGGAGGCCAGTGAATTCCTCTGTGGGTTTTTAGAGG
ADP2R	ATCTGCAGCTCGAGCTCGATGGATCCTTAATAGTCGTTTTGTGAG
ADCPF	ATATGGCCATGGAGGCCAGTGAATTCATGAATTCAGTAGGCCGTA
ADCPR	ATCTGCAGCTCGAGCTCGATGGATCCCTATTTGGGAGTCATGTTG
ADP6F	ATATGGCCATGGAGGCCAGTGAATTCATGGATGATCTACATGTGA
ADP6R	ATCTGCAGCTCGAGCTCGATGGATCCTTAAACCCTTTGAATGTTG
ADSRC2F	ATATGGCCATGGAGGCCAGTGAATTCATGGCAACTGGTTCTTCT
ADSRC2R	ATCTGCAGCTCGAGCTCGATGGATCCATATTCAACACTGTAATT
ADIDD2F	ATATGGCCATGGAGGCCAGTGAATTCATGGCAGAGATGGAAAAT
ADIDD2R	ATCTGCAGCTCGAGCTCGATGGATCCAAGCATAGAAGGCTTTCT
ADPPD5F	ATATGGCCATGGAGGCCAGTGAATTCATGGCTACAGCTCTGCTC
ADPPD5R	ATCTGCAGCTCGAGCTCGATGGATCCCCAAAACCTCCAGGGATC
ADFBA1F	ATATGGCCATGGAGGCCAGTGAATTCATGGCCTCAGCATCTCTA
ADFBA1R	ATCTGCAGCTCGAGCTCGATGGATCCGTAAACATAGCCTTTTAC
ADRABG3F	ATATGGCCATGGAGGCCAGTGAATTCATGCCTTCACGCCGGCGA
ADRABG3R	ATCTGCAGCTCGAGCTCGATGGATCCACACTCGCATCCACCTGT
ADILR1F	CGCGAATTCATGGATTTCTCCAGATG
ADILR1R	CGCGGATCCTTACAATTCATCGTGATG
ADPSMA7F	ATATGGCCATGGAGGCCAGTGAATTCATGGCTAGATATGATAGA
ADPSMA7R	ATCTGCAGCTCGAGCTCGATGGATCCGGTTTCTTTGGGGGCCTT
ADCAT1F	ATATGGCCATGGAGGCCAGTGAATTCATGGATCTCTCTAAGTTT
ADCAT1R	ATCTGCAGCTCGAGCTCGATGGATCCCATTGTAGGCTTTAGAGT
ADAEPF	ATATGGCCATGGAGGCCAGTGAATTCATGGAGTTTTCTGGATCA
ADAEPR	ATCTGCAGCTCGAGCTCGATGGATCCGCCCTGGGCGAAGTTCCT
ADPRS6BF	ATATGGCCATGGAGGCCAGTGAATTCATGGCAACACCAATGGTT
ADPRS6BR	ATCTGCAGCTCGAGCTCGATGGATCCCTTGTAAAACTCAAAATC
ADNF-YC1F	ATATGGCCATGGAGGCCAGTGAATTCATGGAAAACAACCAGCAA
ADNF-YC1R	ATCTGCAGCTCGAGCTCGATGGATCCACTTTGACCATCAAGGTT
ADSnRK2F	ATATGGCCATGGAGGCCAGTGAATTCATGGGGAATGTTAATGGA
ADSnRK2R	ATCTGCAGCTCGAGCTCGATGGATCCCCTCTGTATGGACTTGTA
ADBH0283F	ATATGGCCATGGAGGCCAGTGAATTCATGGCCATGAAACCCGTG
ADBH0283R	ATCTGCAGCTCGAGCTCGATGGATCCTACTAGAAGAGAACCTTC
ADzingipain-1F	ATATGGCCATGGAGGCCAGTGAATTCATGGCTTTGAAAATTTGC
ADzingipain-1R	ATCTGCAGCTCGAGCTCGATGGATCCAGCAGTAGGGAAAGAAGC
ADPORF	ATATGGCCATGGAGGCCAGTGAATTCATGGCTCTTCAGGCTGCT
ADPORR	ATCTGCAGCTCGAGCTCGATGGATCCAGCCAAACCGACGAGTTT
ADSnRK1F	ATATGGCCATGGAGGCCAGTGAATTCATGGGGAATGCGAACGGA
ADSnRK1R	ATCTGCAGCTCGAGCTCGATGGATCCCCTCTTCAGTGGCTTGTA
ADGATA5F	ATATGGCCATGGAGGCCAGTGAATTCATGCTTTACCGAACTCAA
ADGATA5R	ATCTGCAGCTCGAGCTCGATGGATCCAAAACTCTGAACCGGCTG

ADRuBisCOF	TGGCATATGATGGCTTCCTCAGTTATG
ADRuBisCOR	GCCGGATCCTTAGTAGCCTTCTGGCTT
ADCO5F	ATATGGCCATGGAGGCCAGTGAATTCATGGGCATATTGAGAGGC
ADCO5R	ATCTGCAGCTCGAGCTCGATGGATCCAAACGAAGGAACGACGCC
ADBFN2F	ATATGGCCATGGAGGCCAGTGAATTCATGAGCTCTCTACAAGGG
ADBFN2R	ATCTGCAGCTCGAGCTCGATGGATCCTATCCAGTTTCGTTTGGA
ADSPPTF	ATATGGCCATGGAGGCCAGTGAATTCATGGGGAAAGGCGCCGCC
ADSPPTR	ATCTGCAGCTCGAGCTCGATGGATCCTCCATTAGATTCTCTCTC
ADDI19F	GCCGAATTCATGGATGCTGATTCATGGA
ADDI19R	GCCGGATCCTCATAAATTATCATCCAGAAT
ADGRP3F	ATATGGCCATGGAGGCCAGTGAATTCATGGGTTACAAGGCATTT
ADGRP3R	ATCTGCAGCTCGAGCTCGATGGATCCTCCATGAATGGCCTCACC
ADBRG2F	ATCGAATTCATGGCTCTTCCTCATCACC
ADBRG2R	TCAGGATCCCTATATGTAAACTTCCATG
ADGSF	ATATGGCCATGGAGGCCAGTGAATTCATGGCTCAGATCTTGGCT
ADGSR	ATCTGCAGCTCGAGCTCGATGGATCCAACATTCAATGCGAGTTT
ADPTSTF	ATATGGCCATGGAGGCCAGTGAATTCATGAAGTCCTTAAATATG
ADPTSTR	ATCTGCAGCTCGAGCTCGATGGATCCTTCCACAATCAATAAATT
ADOTU1F	ATATGGCCATGGAGGCCAGTGAATTCATGGAAGGTGCTATAGTT
ADOTU1R	ATCTGCAGCTCGAGCTCGATGGATCCTTTGAACTCCTGGAAGTT
BPSnRK1F	GGGGACAAGTTTGTACAAAAAAGCAGGCTTCATGGGGAATGCGAACGGA
BPSnRK1R	GGGGACCACTTTGTACAAGAAAGCTGGGTCCCTCTTCAGTGGCTTGTA
BPRuBisCOF	GGGGACAAGTTTGTACAAAAAAGCAGGCTTCATGGCTTCCTCAGTTATG
BPRuBisCOR	GGGGACCACTTTGTACAAGAAAGCTGGGTCGTAGCCTTCGGGCTTGTA
BPGSF	GGGGACAAGTTTGTACAAAAAAGCAGGCTTCATGGCTCAGATCTTGGCT
BPGSR	GGGGACCACTTTGTACAAGAAAGCTGGGTCAACATTCAATGCGAGCTT
BPFBA1F	GGGGACAAGTTTGTACAAAAAAGCAGGCTTCATGGCCTCAGCATCTCTA
BPFBA1R	GGGGACCACTTTGTACAAGAAAGCTGGGTCGTAGACATAGCCTTTTAC
BPCO5F	GGGGACAAGTTTGTACAAAAAAGCAGGCTTCATGGGCATATTGAGAGGC
BPCO5R	GGGGACCACTTTGTACAAGAAAGCTGGGTCAAAACGAAGGAACGACGC
BPPPD5F	GGGGACAAGTTTGTACAAAAAAGCAGGCTTCATGGCTACAGCTCTGCTC
BPPPD5R	GGGGACCACTTTGTACAAGAAAGCTGGGTCCCAAAACCTCCAGGGATC
BPCAT1F	GGGGACAAGTTTGTACAAAAAAGCAGGCTTCATGGATCTCTCTAAGTTT
BPCAT1R	GGGGACCACTTTGTACAAGAAAGCTGGGTCCATTGTAGGCTTTACAGT
BPPRS6BF	GGGGACAAGTTTGTACAAAAAAGCAGGCTTCATGGCAACACCAATGGTT
BPPRS6BR	GGGGACCACTTTGTACAAGAAAGCTGGGTCCTTGTAGAACTCAAAATC
BPNF-YC1F	GGGGACAAGTTTGTACAAAAAAGCAGGCTTCATGGAAAACAACCAGCAA
BPNF-YC1R	GGGGACCACTTTGTACAAGAAAGCTGGGTCACTTTGACCATCAAGGTT
BPSnRK2F	GGGGACAAGTTTGTACAAAAAAGCAGGCTTCATGGGGAATGTTAATGGA
BPSnRK2R	GGGGACCACTTTGTACAAGAAAGCTGGGTCCCTCTGTATGGACTTGTA
BPPORF	GGGGACAAGTTTGTACAAAAAAGCAGGCTTCATGGCTCTTCAGGCTGCT
BPPORR	GGGGACCACTTTGTACAAGAAAGCTGGGTCAGCCAAACCGACGAGTTT
BPSRC2F	GGGGACAAGTTTGTACAAAAAAGCAGGCTTCATGGCAACTGGTTCTTCT
BPSRC2R	GGGGACCACTTTGTACAAGAAAGCTGGGTCATATTCAACACTGTAATT
BPAEPF	GGGGACAAGTTTGTACAAAAAAGCAGGCTTCATGGAGTTTTCTGGATCA
BPAEPR	GGGGACCACTTTGTACAAGAAAGCTGGGTCGCCTTGGGCGAAGTTCCT
QRT-BYDV-CPF	GTGCACACAATCAACCTTGG
QRT-BYDV-CPR	CCGGCGGTATCAGATGTACT
QRT-CO5F	AGATGGGAGCTCAAATGCAG
QRT-CO5R	ATTCTTGGCCTTGTTTCAGC
QRT-PORF	AAAGCAAACCTTGGGGACTT
QRT-PORR	GTGGTATCGCCTGTGGAACT
QRT-RuBisCOF	TTCGTCTACCGTGAACACCA

QRT-RuBisCOR	GTGGGTAAGCCTTCTTTGCC
QRT-SnRK1F	ACAAGGTTTGCTGGGAGATG
QRT-SnRK1R	CGTCAGACCAAGAGCAACAA
QRT-SnRK2F	ACCTCCTCAGTCCCCAGATT
QRT-SnRK2R	TCTCGAGAGAGGAGGTGGAA
QRT-NF-YC1F	ACAGCTAGTGGTGTGCCTTA
QRT-NF-YC1R	TGCCTCCACTTGCATAGGAA
QRT-CAT1F	ATCGCGATGAAGAGGTGGAT
QRT-CAT1R	CCTGCCTGCTTGAAGTTGTT
QRT-PPD5F	GGCAAGCAATGGGACAGAAT
QRT-PPD5R	CTCCAGGGATCCTTGTACGG
QRT-BRG2F	CGAATGGAACAGTTGGCTTT
QRT-BRG2R	TACAGCACGATGCAGTGTCA
QRT-AEPF	GGATGACAGCACAGGACAAC
QRT-AEPR	TTCCACTGCGTCATTTCGAC
QRT-NbUBCF	TTTCGGTCCTGATGATACTCCC
QRT-NbUBCR	CACAGAGCAAAGACTGGATTGA
